# Osteoporotic fractures and obesity affect frailty progression: a longitudinal analysis of the Canadian multicentre osteoporosis study

**DOI:** 10.1186/s12877-017-0692-0

**Published:** 2018-01-05

**Authors:** Olga Gajic-Veljanoski, Alexandra Papaioannou, Courtney Kennedy, George Ioannidis, Claudie Berger, Andy Kin On Wong, Kenneth Rockwood, Susan Kirkland, Parminder Raina, Lehana Thabane, Jonathan D. Adachi, For the CaMos Research Group, Alexandra Papaioannou, David Goltzman, Nancy Kreiger, Alan Tenenhouse, Elham Rahme, J. Brent Richards, Suzanne N. Morin, Suzanne Godmaire, Silvia Dumont, Claudie Berger, Carol Joyce, Christopher S. Kovacs, Minnie Parsons, Susan Kirkland, Stephanie M. Kaiser, Barbara Stanfield, Jacques P. Brown, Louis Bessette, Jeanette Dumont, Martin Després, Tassos P. Anastassiades, Tanveer Towheed, Wilma M. Hopman, Karen J. Rees-Milton, Robert G. Josse, Angela M. Cheung, Barbara Gardner-Bray, Jonathan D. Adachi, Alexandra Papaioannou, Shannon Reitsma, Wojciech P. Olszynski, K. Shawn Davison, Jola Thingvold, David A. Hanley, Steven K. Boyd, Jane Allan, Jerilynn C. Prior, Shirin Kalyan, Brian Lentle, Millan S. Patel, Bernice Liang, Stuart D. Jackson, William D. Leslie

**Affiliations:** 1 Department of Medicine, McMaster University, Hamilton, ON Canada; 2 Hamilton Health Sciences–Geriatric Education and Research in Aging Sciences (GERAS) Centre, 88 Maplewood Ave, Hamilton, ON L8M 1W9 Canada; 3 St. Joseph’s Healthcare Hamilton, Hamilton, ON Canada; 4 CaMos – McGill University, Montreal, Québec, Canada; 5 Osteoporosis and Women’s Health Program, University Health Network, Toronto, ON Canada; 6 Dalhousie University, Halifax, NS Canada

**Keywords:** Frailty, Fractures, Obesity, Changes over time, Longitudinal analysis

## Abstract

**Background:**

Despite knowing better how to screen older adults, understanding how frailty progression might be modified is unclear. We explored effects of modifiable and non-modifiable factors on changes in frailty in community-dwelling adults aged 50+ years who participated in the Canadian Multicentre Osteoporosis Study (CaMos).

**Methods:**

Rates of change in frailty over 10 years were examined using the 30-item CaMos Frailty Index (CFI). Incident and prevalent low-trauma fractures were categorized by fracture site into hip, clinical vertebral and non-hip-non-vertebral fractures. Multivariable generalized estimating equation models accounted for the time of frailty assessment (baseline, 5 and 10 years), sex, age, body mass index (BMI, kg/m^2^), physical activity, bone mineral density, antiresorptive therapy, health-related quality of life (HRQL), cognitive status, and other factors for frailty or fractures. Multiple imputation and scenario analyses addressed bias due to attrition or missing data.

**Results:**

The cohort included 5566 women (mean ± standard deviation: 66.8 ± 9.3 years) and 2187 men (66.3 ± 9.5 years) with the mean baseline CFI scores of 0.15 ± 0.11 and 0.12 ± 0.10, respectively. Incident fractures and obesity most strongly predicted frailty progression in multivariable analyses. The impact of fractures differed between the sexes. With each incident hip fracture, the adjusted mean CFI accelerated per 5 years by 0.07 in women (95% confidence interval [CI]: 0.03 to 0.11) and by 0.12 in men (95% CI: 0.08 to 0.16). An incident vertebral fracture increased frailty in women (0.05, 95% CI: 0.02 to 0.08) but not in men (0.01, 95% CI: -0.07 to 0.09). Irrespective of sex and prevalent fractures, baseline obesity was associated with faster frailty progression: a 5-year increase in the adjusted mean CFI ranged from 0.01 in overweight (BMI: 25.0 to 29.9 kg/m^2^) to 0.10 in obese individuals (BMI: ≥ 40 kg/m^2^). Greater physical activity and better HRQL decreased frailty over time. The results remained robust in scenario analyses.

**Conclusions:**

Older women and men with new vertebral fractures, hip fractures or obesity represent high-risk groups that should be considered for frailty interventions.

**Electronic supplementary material:**

The online version of this article (10.1186/s12877-017-0692-0) contains supplementary material, which is available to authorized users.

## Background

The segment of the population aged 60 years or older is the fastest growing. It is expected to double by 2050 (from 901 million in 2015 to 2.1 billion), representing 22% of the global population [1]. However, longevity may not be associated with healthy aging, but with frailty [2, 3]. Frailty results from the accumulation of age-related deficits in different physiological systems and is a clinical state that leads to greater risks of adverse health outcomes, such as falls, fractures, hospitalizations, loss of independence, and death [4–8]. Several screening tools for frailty have also been validated [9, 10]. However, understanding how frailty progression may be modified remains unclear. Cross-sectional studies have demonstrated the impact of risk factors such as physical activity [6–8]; but, a gap persists regarding important modifiable and non-modifiable predictors of frailty change over time. We explored the effects of low-trauma fractures, obesity and other modifiable and non-modifiable factors on changes in frailty over time in Canadians aged 50 years and older.

## Methods

### Setting and study population

The Canadian Multi-centre Osteoporosis Study (CaMos) is a population-based study primarily designed to delineate the impact and prevention of osteoporosis in Canada [11]. A random age-, sex-, and region-specific community-dwelling sample of 9423 adults (71% women), aged 25 years and older, able to communicate in English, French or Chinese was recruited in 1995/6 across seven provinces and nine urban and rural cities [11]. Over 60% were followed prospectively for 20 years (until 2016), and examined comprehensively every 5 years using questionnaires and in-person clinical assessments, bone mineral density (BMD) tests and radiographs [12]. We conducted a longitudinal analysis of the currently accessible 10-year data for a cohort of women and men aged 50 years and older, regardless of their history of fracture. Ethics approval was granted through the Research Ethics Board of academic institutions associated with each CaMos centre.

### Main exposure and covariates

Prevalent clinical fractures were reported at baseline and incident fractures were reported at annual follow-ups and confirmed by structured interviews via telephone or in-person [12]. The questionnaires included information related to fracture site, fracture number, circumstance, treatment, and radiographs or medical reports. Incident fractures were defined as any new low-trauma fracture excluding head, toe and finger fractures. Our main analyses categorized low-trauma fractures by location, which included hip, clinical vertebral, and non-hip non-vertebral (NHNV) fractures (e.g., leg not hip, pelvis, rib, shoulder, scapula, upper arm, wrist/forearm, and hand). We also examined the effects of prevalent (prior to baseline) and new single or multiple fractures where multiple fractures represented more than one event of any clinical low-trauma fracture (same or different type). Incident fractures were analyzed as time-varying predictors accounting for the presence of a new event in two time periods, between years 1 and 5 and years 6 and 10. Individuals without fractures represented the reference group.

All analyses were adjusted for baseline age and time of frailty assessment. We categorized age into three groups: 50–<65[reference], 65–<80 and ≥80 years; the time variable represented the duration of follow-up at which frailty was measured: baseline [reference, 1996], years 5 and 10 (2006). We explored the effects of sex and baseline body mass index (BMI, in kg/m^2^) on changes in frailty. BMI was categorized as: underweight (<18.49), normal weight (18.5–24.99, reference), overweight (25.0–29.9), obese class I/II (30.0–39.9), and pathologically obese-class III (≥ 40.0) [13]. Our models also included: 1) socio-demographic factors: ethnicity (Caucasian vs. other), education (university or higher degrees vs. no university), employment history (employed full time or part time [reference], retired, homemaker, unemployed), and living arrangement (living alone: yes/no); 2) anthropometrics and lifestyle: excessive weight loss (> 10 pounds), physical activity related to strenuous, vigorous or moderate exercise reported in kilocals/week (changes in frailty analyzed per 1000 kilocals/week, equivalent to 3–6 METs [14, 15]), sedentary lifestyle (hours/day), smoking (never, past and current[reference]), and daily alcohol consumption (≥3 drinks, 1–<3, >0 to <1, and none); 3) bone health: femoral neck BMD T-score, history of falls (past month: yes/no), bed rest (immobilization: yes/no), antiresorptive therapy (baseline: yes/no), and total daily calcium and vitamin D intakes from food and supplements (changes in frailty analyzed per 1200 mg/day and 800 IU/day, respectively); 4) health-related quality of life (HRQL) measured by the physical and mental health subscales of the Medical Outcomes Trust SF-36 Health Survey (changes in frailty analyzed per a 5-point change in SF-36 scores); 5) cognitive status measured by the Mini Mental State Examination (MMSE), assessed in participants aged 65+ (changes in frailty analyzed per a 3.72-point change in MMSE scores). All analyses were also adjusted for the use of antiresorptives at year 10 due to substantial changes in their availability over time (1996: etidronate; 2006: etidronate, alendronate, clodronate, risedronate, pamidronate, zoledronate).

### Outcome

Frailty was measured by the 30-item CaMos Frailty Index (CFI). The construction and validation of the CFI is described in detail elsewhere [16]. In brief, it was developed in the CaMos cohort aged 25 to 103 years (*N* = 9423) using a cumulative deficits framework [17]. It included 30 variables related to a wide range of deficits in biologic systems (e.g., signs, symptoms, disease states and disabilities) that accumulated but did not saturate quickly with age, and had <5% of missing data [16]. Thus, it included the following comorbidities: osteoarthritis, rheumatoid arthritis, thyroid disease, breast cancer, uterine/prostate cancer, inflammatory bowel disease, hypertension, heart disease (e.g., heart attack), stroke, thrombophlebitis, neuromuscular disease, diabetes type 1 or type 2, and kidney disease. It also included variables related to: general health, change in general health, feelings of having energy and tiredness, as well as deficits in: vision, hearing, walking, dexterity, cognition, pain, daily work, social activities, and limitations in: in moderate activities (e.g., moving table, vacuuming, golf, bowling), lifting or carrying groceries, climbing a flight of stairs, bending, kneeling, stooping, bathing or dressing (Additional file 1). Total CFI scores ranged from 0 to 1, with higher values indicating greater frailty; the upper limit was 0.66 and the mean rate of deficit accumulation per year of age was 0.04 (i.e., a minimal clinically important difference) [16].

### Statistical analysis

In descriptive analyses, categorical variables were expressed as percentages and continuous data by means and standard deviations (SD). Generalized estimating equations models with an autoregressive correlation structure were used to analyze repeated measurements and associations between rates of change in frailty over time and predictors. Regression estimates generated in unadjusted and adjusted analyses indicated increases or decreases in the mean CFI score per 5 years (i.e., rates of change in frailty) for a unit change in a predictor (e.g., each new fracture). All models were adjusted for a statistically significant age-time interaction (*p* < 0.0001) indicating differences in changes in frailty for different age groups. We also confirmed the modifying effects of sex (*p* = 0.0005), prevalent hip (*p* = 0.006) and clinical vertebral fractures (*p* = 0.03), and developed the following five models: one for each sex for the whole sample (women, *n* = 5566; men, *n* = 2187), one for each sex for the sample without prior fractures (women, *n* = 4348; men, *n* = 1814), and one for the participants with prior fractures (*n* = 1574) as the rate of change was not significantly different between women and men with prior fractures (*p* = 0.62). We examined bias due to missing data or attrition using multiple imputations and worst-case scenarios. In the worst-case scenarios, for participants dropping out at years 5 and 10, we imputed the highest CFI value estimated for hip fractures (i.e., CFI = 0.268) or the highest upper limit reported in the literature (CFI = 0.70) [17]. Statistical significance was set at an alpha-level of 0.05. Analyses were performed using SAS 9.4 (SAS Institute, Inc., NC). Additional results are presented in Additional file 2.

## Results

The cohort included 7753 CaMos participants (5566 women) aged 50 years and older. Of these, 6162 participants (4348 women) reported no fractures, and 1574 participants had prior (prevalent) low-trauma fractures (1206 women). Table 1 presents their demographic, anthropometric, and lifestyle characteristics, comorbidities, bone health, HRQL and cognitive status. The mean baseline age (±SD) for all participants was 66.7 ± 9.4 years (women: 66.8 ± 9.3; men: 66.3 ± 9.5); for those without prevalent fractures, it was 66.2 ± 9.3 years, and for those with prior fractures, it was 68.9 ± 9.2 years. Participants aged 80+ comprised 9.0% of the sample without prior fractures and 11.4% of the sample with prior fractures. The mean baseline BMI was 27.1 kg/m^2^ and at least one in five participants was obese (class I to III). Participants expended on average 4160 to 4900 kcal weekly on exercise, the majority including moderate physical activities such as brisk walking. Also, 34% of participants without prior fractures and 41% of those with prior fractures reported up to four comorbidities at baseline. Approximately 20% of the participants used antiresorptives at baseline, and up to 36% used them at year 10.

**Table 1:** Baseline characteristics of the examined cohorts

Baseline characteristics	Whole sample			No prior fracture			With prior fracture (*N* = 1574)		
	Total (*N* = 7753)^a^	Women (N = 5566)	Men (N = 2187)	Total (*N* = 6162)	Women (N = 4348)	Men (N = 1814)	Total (*N* = 1574)^b^	Women (*N* = 1206)	Men (*N* = 368)
Demographic characteristics									
Age, years: Mean (SD)	66.7 (9.4)	66.8 (9.3)	66.3 (9.5)	66.2 (9.3)	66.2 (9.2)	66.2 (9.5)	68.4 (9.3)	68.9 (9.2)	66.6 (9.4)
Age group: n (%)									
50 to <65	3205 (42)	2263 (41)	942 (43)	2678 (43)	1892 (43)	780 (43)	523 (33.2)	369 (30.6)	154 (41.9)
65 to <80	3828 (49)	2780 (50)	1048 (48)	2947 (48)	2080 (48)	867 (48)	872 (55.4)	693 (57.5)	179 (48.6)
≥ 80	72 (9)	523 (9)	197 (9)	537 (9)	376 (9)	161 (9)	179 (11.4)	144 (11.9)	35 (9.5)
Females: n (%)	5566 (72)	5566 (72)	0 (0)	4348 (71)	4348 (100)	0 (0)	1206 (77)	1206 (100)	0 (0)
Education (some university or higher): n (%)	1899 (24)	1166 (21)	733 (33)	1476 (24)	871 (20)	605 (33)	421 (26.8)	295 (24.5)	126 (34.2)
Caucasians: n (%)	7405 (96)	5361 (96)	2044 (93)	5852 (95)	4164 (96)	1688 (93)	1536 (97.6)	1185 (98.3)	351 (95.4)
Employment: n (%)									
Employed (FT, PT)	1711 (22)	1087 (19)	624 (28)	1426 (23)	911 (21)	515 (28)	282 (17.9)	175 (14·5)	107 (29.1)
Homemaker	1433 (18)	1431 (26)	2 (1)	1121 (18)	1119 (26)	2·0 (0.1)	306 (19.4)	306 (25.4)	0 (0)
Retired	4250 (55)	2826 (51)	110 (65)	3321 (54)	2136 (49)	1185(65)	921 (58.5)	685 (56·8)	236 (64.1)
Unemployed, disability, other	357 (5)	220 (4)	137 (6)	292 (5)	180 (4)	112 (6)	65 (4.2)	40 (3·3)	25 (6.8)
Living alone: n (%)	2573 (33)	2069 (37)	504 (23)	1939 (31)	1528 (35)	411 (23)	630 (40)	538 (44·6)	92 (25)
Anthropometric and lifestyle factors									
Body Mass Index [BMI], kg/m^2^: Mean (SD)	27.1 (4.8)	27.0 (5.1)	27.2 (4.0)	27.1 (4.7)	27.0 (5.0)	27.2 (4.0)	27.1 (4.9)	27.1 (5.2)	27.0 (3.8)
Body Mass Index (kg/m^2^): number (%)									
Underweight: BMI < 18.5	130 (2)	112 (2)	18 (1)	90 (2)	74 (2)	16 (0.9)	40 (2.6)	21 (1.8)	2 (1)
Normal weight: BMI: 18.5 to <25.0	2529 (34)	1926 (36)	603 (28)	2055 (34)	1549 (37)	506 (28·7)	470 (31)	267 (23)	97 (26)
Overweight: BMI: 25.0 to <30.0	3115 (41)	2046 (38)	1069 (50)	2456 (41)	1582 (37)	874 (49·5)	652 (43)	460(40)	192(52)
Obesity, class I-III: 30.0 to <40.0	1644 (22)	1217 (23)	427 (20)	1307 (22)	947 (22)	360 (20·4)	332 (22)	373 (32)	65 (18)
Obesity, class IV: ≥ 40.0	106 (1)	93 (1)	13 (1)	82 (1)	72 (2)	10 (0·6)	24 (1.6)	38(3)	3(1)
Ever lost 10 pounds: number (%)	3897 (50)	2854 (51)	1043 (48)	3036 (49)	2193 (50)	843 (41)	850 (54)	653 (54)	197 (54)
Physical activity, all exercise (kilocal/week): Mean (SD)	4354.6 (3634.9)	4123.4 (3156.3)	4937.8 (4581.8)	4402.2 (3599.1)	4187.5 (3150.9)	4917.3 (4454.3)	4163 (3780)	3887.4 (3174)	5053 (5191)
Strenuous exercise- jogging, bicycling, tennis, swimming (kilocal/week): Mean (SD)	304.5 (1134.6)	222.5 (842.5)	512·5 (1640.9)	306·7 (1105.7)	229.3 (846.6)	492·4 (1545.1)	298.1 (1247.3)	198.4 (830.4)	619.9 (2056)
Vigorous exercise -moving heavy furniture, shoveling, weight lifting (kilocal/week):									
Mean (SD)	411.3 (1798.7)	196.5 (1042.0)	956.5 (2877.5)	432.2 (1831.5)	206.1 (1026.1)	974.4 (2908.6)	330.6 (1669)	163.1 (1103)	871.7 (2736)
Moderate exercise- housework, brisk walking, golfing, bowling, gardening (kilocal/week):									
Mean (SD)	3637.7 (2894.8)	3704.2 (2812.7)	3468.9 (3087.7)	3663.2 (2903.5)	3751.8 (2834.4)	3450.8 (3053.1)	3534.4 (2866.6)	3525.9 (2731.8)	3561.8 (3268.5)
Smoking: n (%)									
Non-smoker	3590 (46)	2956 (53)	634 (29)	2837 (46)	2314 (53)	523 (29)	746 (47)	636 (53)	110 (30)
Past smoker	3054 (39)	1855 (33)	119 (55)	2450 (40)	1450 (33)	1000(55)	597(38)	402(33)	195(53)
Current smoker	1107 (14)	754 (14)	353 (16)	874 (14)	583 (14)	291 (16)	230 (15)	168 (14)	62 (17)
Alcohol consumption, drinks per year	150.4 (304.9)	102.1 (211.8)	273.3 (441.0)	151.7 (306.8)	100.4 (210.7)	274.5 (438)	145.2 (296.7)	108.6 (216.2)	265.4 (453)
Alcohol consumption per day: n(%)									
None	3296 (42)	2643 (48)	653 (30)	2601 (42.2)	2064 (47.5)	537(29.6)	687(43.7)	572(47.3)	115(31.3)
0–1	3082 (40)	2204 (40)	878 (40)	2452 (39.8)	1724 (39.7)	728(40.1)	625(39.7)	477(39.6)	148(40.2)
1–3	1176 (15)	674 (12)	502 (23)	953 (15.5)	533 (12.3)	420 (23.2)	220 (14.0)	139 (11.5)	81 (22.0)
≥ 3	199 (3)	45 (1)	154 (7)	156 (2.5)	27 (0.6)	129 (7.1)	42 (2.7)	18 (1.5)	24 (6.5)
Bone and overall health									
BMD T-score at the femoral neck, baseline: Mean (SD)	−1.5 (1.0)	−1.6 (1.0)	−1.2 (0.8)	−1.4 (1.0)	−1.6 (1.0)	−1.2 (0.8)	−1.8 (1.0)	−2.0 (1.0)	−1.4 (0.85)
Baseline low trauma fracture, excluding head, toe and finger fractures: n (%)	1574 (20)	1206 (22)	368 (17)	NA	NA	NA	1574 (100)	1206 (100)	368 (100)
Mean number of comorbidities^c^, Mean (SD)	1.3 (1.3)	1.5 (1.3)	1.3 (1.3)	1.3 (1.3)	1.4 (1.3)	1.1 (1.1)	1.5 (1.4)	1.6 (1.4)	1.2 (1.1)
Comorbidities^c^, count: n (%)									
None	2289 (29)	1476 (27)	813 (37)	1889 (31)	1197 (27)	692 (38)	395 (25)	277 (23)	118 (32)
1	2531 (33)	1785 (32)	746 (34)	2039 (33)	1422 (33)	617 (34)	488 (31)	359 (30)	129 (35)
2–4	2742 (35)	2136 (38)	606 (28)	2094 (34)	1606 (37)	488 (27)	640 (41)	524 (43)	116 (32)
≥ 5	191 (3)	169 (3)	22 (1)	140 (2)	123 (3)	17 (1)	51 (3)	46 (4)	5 (1)
Falls (past month): n (%)	503 (6.0)	356 (7.0)	138 (6.0)	379 (6.2)	272 (6.4)	107 (5.9)	124 (7.9)	93 (7.7)	31 (8.4)
Immobilized: n(%)	1049 (13)	754 (14)	295 (13)	702 (11)	485 (11)	217 (12)	345 (21.9)	267 (22)	78 (21)
Calcium intake, food & supplements (mg/day): Mean (SD)	1020.6 (617.8)	1062.1 (625.8)	913.9 (583.2)	1005.1 (611.5)	1048.6 (622.2)	899.9(571.7)	1081.8 (639.0)	1111.7 (637.6)	983.4 (636.6)
Vitamin D, food and supplements (IU/day): Mean (SD)	298.1 (1003.4)	331.9 (1157.0)	211.8 (387.0)	276.8 (883.1)	305.2 (1027.2)	208.8 (334.0)	382.0 (1378)	429.9 (1537.0)	224.6 (582.1)
Use of antiresorptive drugs, baseline: n(%)	1527 (20)	1522 (27)	5 (0·2)	1217 (20)	1214 (28)	3 (0.2)	308 (19·6)	306 (25·4)	2 (0.54)
Use of antiresorptive drugs, year 10: n(%)	1361 (31)	1269 (39)	92 (8)	1059 (30·1)	990 (38.3)	69 (7.4)	301 (36.4)	278 (43)	23 (12.7)
Health-Related quality of life									
SF-36, Physical subscale score: Mean (SD)	46.2 (10.4)	45.7 (10.6)	47.6 (9.7)	46.7 (10.2)	46.3 (10.4)	47.8 (9.7)	44.1 (10.8)	43.4 (11.1)	46.4 (9.6)
SF-36, Mental health subscale score: Mean (SD)	53.8 (8.6)	53.4 (8.9)	54.7 (7.8)	53.7 (8.6)	53.4 (8.8)	54.6 (7.9)	54.0 (8.7)	53.6 (9.2)	55.3 (6.9)
Cognitive status									
MMSE, total score: Mean (SD)	27.8 (2.4)	28.1 (2.3)	27.6 (2.7)	28.0 (2.4)	28.1 (2.3)	27.6 (2.6)	28.1 (2.4)	28.1 (2.2)	27.8 (3.0)

^a^Sample including all reported fractures; ^b^ Sample with low-incident fractures, excluding head, finger and toe fractures; *n* number of participants, *SD* standard deviation; *NA* not applicable; ^c^Comorbidities included in the Canadian Multicentre Osteoporosis Study (CaMos) Frailty Index: osteoarthritis, rheumatoid arthritis, thyroid disease, breast cancer, uterine/prostate cancer, inflammatory bowel disease, hypertension, heart disease, stroke, thrombophlebitis, neuromuscular disease, diabetes type 1 or type 2, and kidney disease; *MMSE* Mini Mental State Examination, assessed in participants age 65+ years

Over 10 years, 893 incident low-trauma fractures occurred in women and 151 in men (Additional file 2: Table S1). They occurred in 7.4% of participants over the first 5 years (women: 8.7%, men: 3.9%) and in 8.1% of participants over the next 5 years (women: 9.5%; men: 4.2%). About 2.0–2.5% of adults reported new hip or clinical vertebral fractures during 10 years (1%: prevalent fractures). A NHNV fracture was the most frequent (12%: prevalent, reported at baseline; 8%: incident). Among NHNV fractures, wrist or forearm fractures were the most frequent (e.g., 44% of all prevalent fractures). Less than 1% of the sample had incident multiple fractures during 10 years.

The mean baseline CFI (± SD) was 0.14 ± 0.11 in participants without prevalent fractures (women: 0.15 ± 0.11, men: 0.12 ± 0.10). It was higher (0.17 ± 0.12) in those with prior fractures (women: 0.18 ± 0.12, men: 0.13 ± 0.10) (Table 2). Changes in frailty over time appeared to be nonlinear: the mean CFI increased on average by 0.03 ± 0.08 over the first 5 years, but it slightly decreased by 0.02 ± 0.08 in the next 5 years. Frailty progression was the greatest in women aged 65+ and men aged 80+ years (Table 3).

**Table 2:** The Canadian Multicentre Osteoporosis Study (CaMos) Frailty Index scores: Baseline, year 5, and year 10

	All participants			Participants with no prior fractures			Participants with prior fractures		
CFI score	Total Mean (SD) [range; n]	Women Mean (SD) [range; n]	Men Mean (SD) [range; n]	Total Mean (SD) [range; n]	Women Mean (SD) [range; n]	Men Mean (SD) [range; n]	Total Mean (SD) [range; n]	Women Mean (SD) [range; n]	Men Mean (SD) [range; n]
Baseline	0.14 (0.11) [0.00–0.66; 7753]	0.15 (0.11) [0.00–0.66; 5566]	0.12 (0.10) [0.00–0.53; 2187]	0.14 (0.11) [0.00–0.66; 6162]	0.15 (0.11) [0.00–0.66; 4348]	0.12 (0.10) [0.00–0.53; 1814]	0.17 (0.12) [0.00–0.60; 1574]	0.18 (0.12) [0.00–0.60; 1206]	0.13 (0.10) [0.00–0.45; 368]
Year 5	0.16 (0.12) [0.00–0.65; 6200]	0.17 (0.12) [0.00–0.65; 4544]	0.14 (0.11) [0.00–0.57; 1656]	0.16 (0.11) [0.00–0.62; 4961]	0.17 (0.12) [0.00–0.61; 3590]	0.13 (0.10) [0.00–0.57; 1371]	0.18 (0.12) [0.00–0.65; 1227]	0.19 (0.12) [0.00–0.65; 945]	0.15 (0.12) [0.00–0.54; 282]
Year 10	0.12 (0.09) [0.00–0.53; 4345]	0.13 (0.10) [0.00–0.53; 3233]	0.11 (0.09) [0.00—0.45; 1112]	0.12 (0.10) [0.00–0.52; 3514]	0.12 (0.10) [0.00–0.52; 2583]	0.10 (0.09) [0.00–0.45; 931]	0.14 (0.10) [0.00–0.53; 827]	0.15 (0.10) [0.00–0.53; 646]	0.11 (0.09) [0.00–0.40; 181]
Absolute change: baseline to year 5	0.03 (0.08) [−0.32–0.43; 6200]	0.03 (0.08) [−0.32–0.37; 4544]	0.03 (0.08) [−0.30–0.43; 1656]	0.03 (0.08) [−0.28–0.43; 4961]	0.03 (0.08) [−0.26–0.37; 3590]	0.03 (0.08) [−0.28–0.43; 1371]	0.03 (0.09) [−0.32–0.43; 1227]	0.03 (0.09) [−0.32–0.32; 945]	0.04 (0.08) [−0.30–0.43; 282]
Absolute change: year 5 to year 10	−0.02 (0.08) [−0.37–0.38; 4303]	−0.02 (0.09) [−0.35–0.38; 4207]	−0.01 (0.08) [−0.37–0.36; 1096]	−0.02 (0.08) [−0.37–0.38; 3481]	−0.02 (0.08) [−0.35–0.38; 2562]	−0.01 (0.08) [−0.37–0.36; 919]	−0.01 (0.09) [−0.33–0.38; 818]	−0.02 (0.09) [−0.32–0.38; 641]	−0.02 (0.07) [−0.33–0.32; 177]

*CFI* CaMos Frailty Index, *SD* standard deviation, *n* sample size

**Table 3:** Changes in the CaMos Frailty Index (CFI) score per a 5-year period in participants aged 50+ years

Parameter			Women (N = 5566)	Men (N = 2187)
			Mean Estimate (95% CI)	Mean Estimate (95% CI)
Time	Year 10		−0.095 (−0.201; 0.010)	−0.008 (−0.010;-0.010)
	Year 5		−0.019 (−0.115;0.078)	−0.050 (−0.050;-0.050)
	Baseline (ref)		0.000	0.000
Age, years	> = 80		−0.005 (−0.073;0.063)	0.061 (0.027;0.095)
	65–80		−0.030 (−0.097;0.037)	0.059 (0.037;0.081)
	50–65 (ref)		0.000	0.000
Time*age	Year 10	> = 80	**0.112 (0.003–0.220)**	**0.078 (0.038;0.118)**
		65–80	0.105 (−0.001;0.210)	**0.029 (0.020;0.037)**
		50–65 (ref)	0.000	0.000
	Year 5	> = 80	0.039 (−0.061;0.138)	**0.100 (0.060;0.139)**
		65–80	0.046 (−0.051;0.143)	**0.082 (0.075;0.089)**
		50–65 (ref)	0.000	0.000
	Baseline (ref)	> = 80	0.000	0.000
		65–80	0.000	0.000
		50–65	0.000	0.000
Prevalent fracture	NHNVF		0.003 (−0.005;0.012)	0.004 (−0.013;0.021)
	Clinical VF		−0.011 (−0.034;0.012)	**0.044 (0.010;0.078)**
	Hip		**0.029 (0.001;0.056)**	0.032 (−0.007;0.070)
	None (ref)		0.000	0.000
Incident fracture	NHNVF		**0.013 (−0.002;0.029)**	0.034 (−0.006;0.073)
	Clinical VF		**0.053 (0.022;0.084)**	0.010 (−0.066;0.086)
	Hip		**0.067 (0.026;0.109)**	**0.121 (0.082;0.161)**
	None (ref)		0.000	0.000
Caucasian, yes			0.003 (−0.011;0.017)	−0.006 (−0.029;0.017)
Body mass index (BMI), kg/m^2^	> = 40		**0.071 (0.040;0.102)**	**0.051 (0.025;0.078)**
	30–40		**0.022 (0.013;0.030)**	**0.020 (0.005;0.036)**
	25–30		**0.008 (0.001;0.014)**	0.010 (−0.002;0.021)
	<18.5		−0.013 (−0.031;0.005)	0.044 (−0.028;0.116)
	18.5–25 (ref)		0.000	0.000
Physical activity (per 1000 kilocals/week)			**−0.002 (−0.002;-0.001)**	0.000 (−0.001;0.001)
Sedentary lifestyle, hours/day			0.001 (0.000;0.002)	**−0.002 (−0.004;0.000)**
Total calcium intake (per 1200 mg/day)			0.003 (−0.003;0.009)	0.002 (−0.009;0.012)
Total vitamin D intake (per 800 /day)			0.000 (−0.001;0.001)	**−0.006 (−0.012;0.000)**
MMSE score (per 3.72 point change)			**−0.010 (−0.017;-0.003)**	−0.004 (−0.017;0.008)
SF-36 - Physical subscale (per 5 unit change)			**−0.031 (−0.032;-0.029)**	**−0.030 (−0.03;-0.03)**
SF-36 - Mental subscale (per 5 unit change)			**−0.015 (−0.016;-0.013)**	**−0.018 (−0.021;-0.014)**
Education: University or higher, yes			−0.003 (−0.010;0.004)	**−0.012 (−0.022;-0.001)**
Smoking	Never		−0.002 (−0.013;0.008)	−0.016 (−0.038;0.006)
	Past		0.010 (0.000;0.021)	−0.005 (−0.026;0.016)
	Current (ref)		0.000	0.000
Alcohol, drinks per day	> = 3		−0.016 (−0.046;0.014)	−0.015 (−0.036;0.006)
	1 to 3		−0.003 (−0.013;0.006)	−0.001 (−0.015;0.013)
	<1 to >0		**−0.007 (−0.013;−0.001)**	0.008 (−0.005;0.020)
	None (ref)		0.000	0.000
Employment	Unemployed		-0.001 (−0.032;0.030)	−0.015 (−0.054;0.025)
	Retired		−0.004 (−0.020;0.013)	−0.009 (−0.023;0.006)
	Homemaker		0.002 (−0.015;0.018)	0·00
	Employed (ref)		0.000	0·00
Living alone, yes			0.003 (−0.003;0.009)	**0.015 (0.002;0.028)**
BMD T-score at baseline			0.002 (−0.001;0.006)	−0.004 (−0.010;0.002)
Antiresorptive treatment at baseline, yes			0.000 (−0.007;0.008)	−0.049 (−0.101;0.002)
Antiresorptive treatment at year 10, yes			0.004 (−0.002;0.010)	**0.025 (0.008;0.042)**
Falls in past month, yes			0.003 (−0.010;0.015)	0.018 (−0.007;0.042)
Ever confined to bed (immobilized), yes			0.000 (−0.010;0.009)	0.010 (−0.004;0.023)
Ever lost more than 10 pounds, yes			0.005 (−0.001;0.010)	0.005 (−0.005;0.015)

*CaMos* the Canadian Multicentre Osteoporosis Study; Estimates are regression coefficients denote the mean change (increase or decrease) in CFI scores per a 5-year period for one unit change/category in a predictor value; *CI* confidence interval, *Ref* the reference group; Bolded font style suggest a statistically significant result at a alpha 0.05 level (p-value <0.05), Time variables represent frailty assessment occasions (waves); The time*age interaction describes changes in frailty for different age groups; *NHNVF* non-hip non-clinical vertebral fractures, *VF* clinical vertebral fractures, *MMSE* Mini Mental State Examination. Note: The estimates were rounded to the third decimal and may appear inexact

**Fig. 1:**
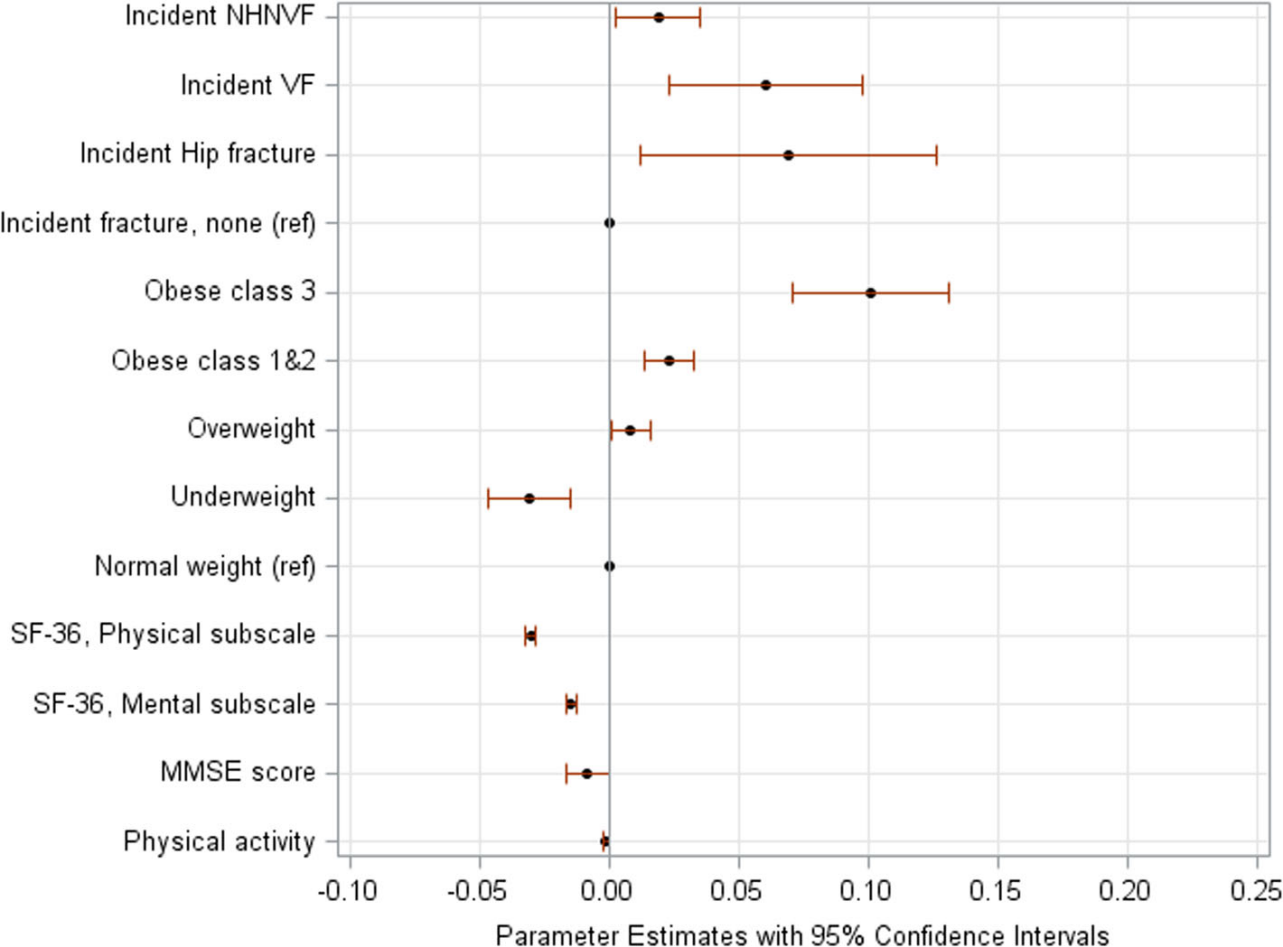
Changes in the Canadian Multicentre Osteoporosis Study Frailty Index (CFI) per a 5-year period in women aged 50+ years without prior low-trauma fracture: Significant predictors. Plots represent statistically significant risk factors shown in the multivariable-adjusted model (Additional file 2: Table S9). Parameter estimates are regression coefficients that denote the mean change (increase: the value >0, and decrease: the value <0) in the CFI score per a 5-year period for one unit change/category in a predictor. NHNVF denotes non-hip-non-clinical vertebral fracture, VF denotes clinical vertebral fracture; ref. denotes the reference group; MMSE denotes Mini Mental State Examination score and the value represents a decrease in frailty over 5 years per a 3.72-point change in the score. The parameter estimate for physical activity denotes a decrease in frailty per 1000 kilocals weekly; for SF-36 scores, decreases in frailty over 5 years are calculated per a 5-point change in the score

**Fig. 2:**
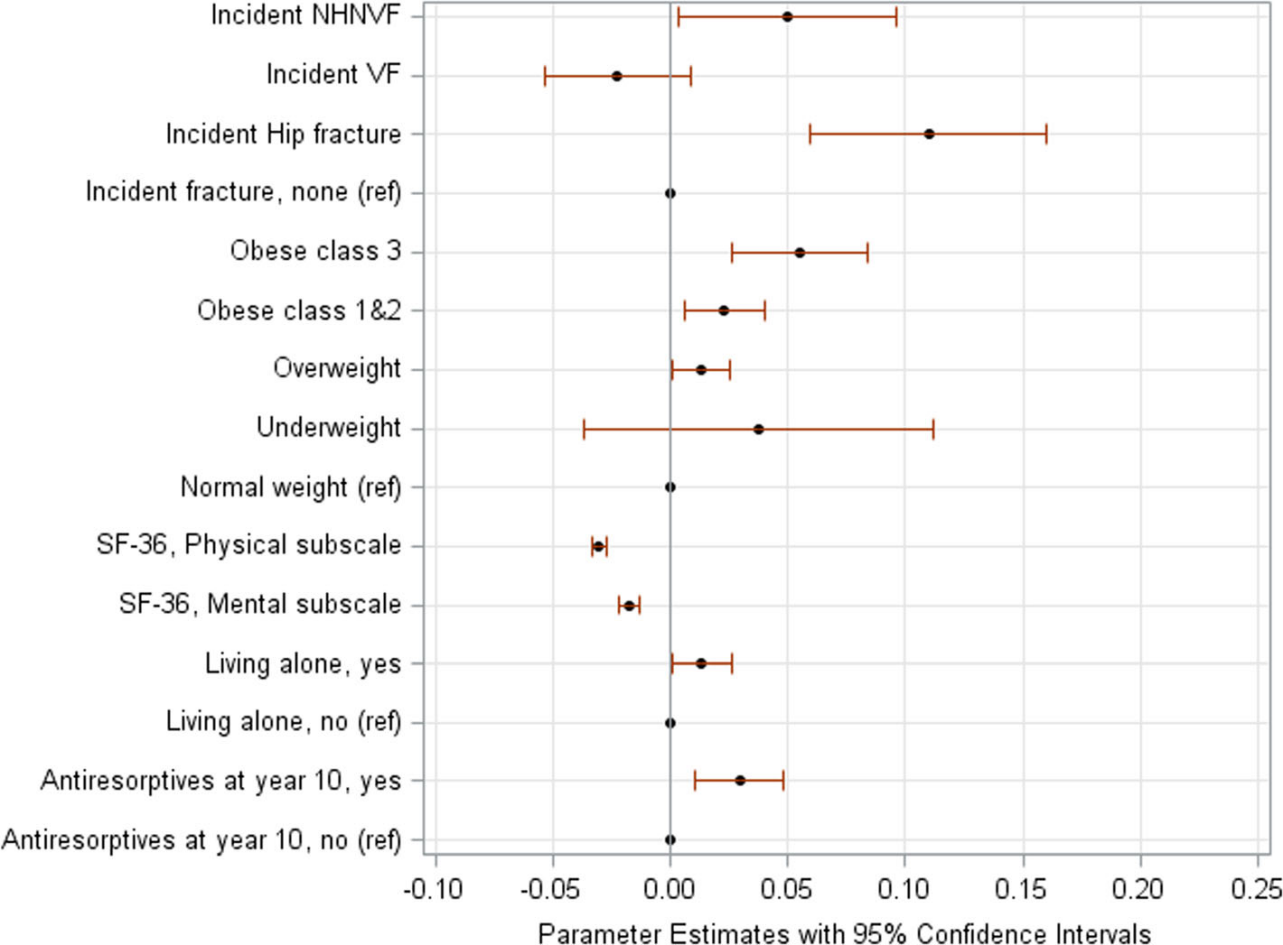
Changes in the Canadian Multicentre Osteoporosis Study Frailty Index (CFI) per a 5-year period in men aged 50+ years without prior low-trauma fracture: Significant predictors. Plots represent risk factors shown statistically significant in the multivariable-adjusted model (Additional file 2: Table S9). Parameter estimates are regression coefficients that denote the mean change (increase: the value >0, and decrease: the value <0) in the CFI score per a 5-year period for one unit change/category in a predictor. NHNVF denotes non-hip-non-clinical vertebral fracture, VF denotes clinical vertebral fracture; ref. denotes the reference group. For SF-36 scores, decreases in frailty over 5 years are calculated per a 5-point change in the score

**Fig. 3:**
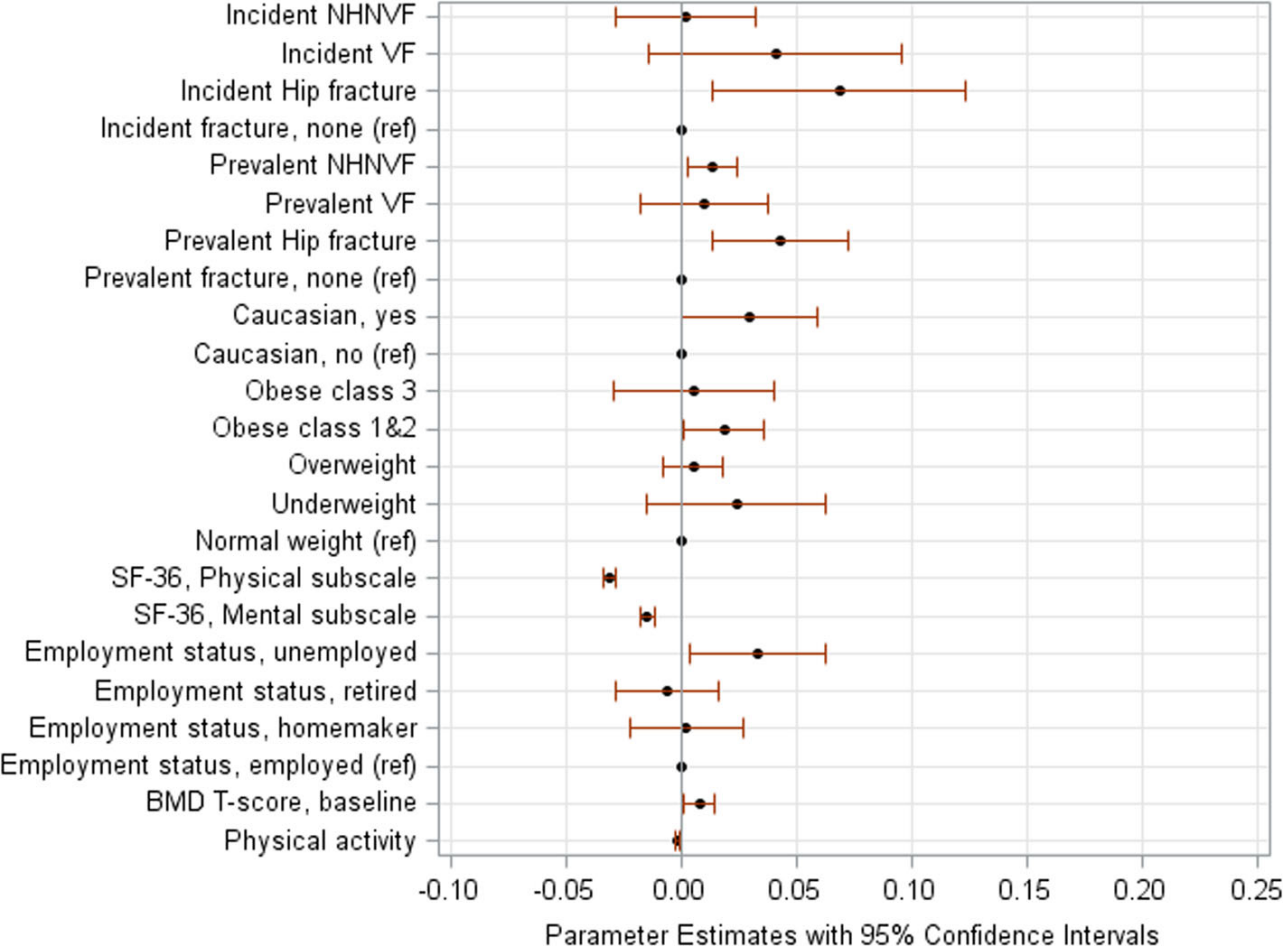
Changes in the Canadian Multicentre Osteoporosis Study Frailty Index (CFI) per a 5-year period in adults aged 50+ years with prior low-trauma fracture: Significant predictors. Plots represent statistically significant risk factors shown in the multivariable-adjusted model (Additional file 2: Table S11). Parameter estimates are regression coefficients that denote the mean change (increase: the value >0, and decrease: the value <0) in the CFI score per a 5-year period for one unit change/category in a predictor. NHNVF denotes non-hip-non-clinical vertebral fracture, VF denotes clinical vertebral fracture; ref. denotes the reference group. The parameter estimate for physical activity denotes a decrease in frailty per 1000 kilocals weekly; for SF-36 scores, decreases in frailty over 5 years are calculated per a 5-point change in the score

In unadjusted and multivariable-adjusted analyses, the progression of frailty was substantially affected by fracture site (Table 3; Additional file 2: Tables S2-S4; S8-S12). The impact differed between two sexes. In the sample including all participants (Table 3 & Additional file 2: Figure S1), the adjusted mean CFI score significantly increased per 5 years after an incident hip fracture by 0.07 in women (95% confidence interval [CI]: 0.03–0.11) and by 0.12 in men (95% CI: 0.08–0.16). An incident clinical vertebral fracture was associated with a similar 0.05 point increase in the adjusted mean CFI score in women (95% CI: 0.02–0.08), and a much smaller increase in men (0.01, 95% CI: -0.07–0.09). Also, incident multiple fractures had larger detrimental effects on frailty progression in women than in men (0.06, 95% CI: 0.01–0.11 vs. 0.03, 95% CI: -0.05–0.06, Additional file 2: Table S8). In women without prior fracture, incident hip and vertebral fractures increased the adjusted mean CFI scores to similar extents (0.07, 95% CI: 0.01–0.13; 0.06, 95% CI: 0.02–0.10); however, in men, incident hip and NHNV fractures variably affected the progression of frailty, increasing the mean score by 0.11 (95% CI: 0.06–0.16) and 0.05 (95% CI: 0.004–0.096) per 5 years, respectively (Figs 1 and 2; Additional file 2: Table S9). In participants with prior fracture, a new hip fracture was the only low-trauma fracture associated with a significantly faster frailty progression (0.07, 95% CI: 0.01–0.12, Fig. 3 and Additional file 2: Table S11).

Baseline BMI was another important predictor of frailty progression. In both women and men, the higher baseline BMI was associated with a larger impact on frailty (Table 3; Additional file 2: Tables S5-S12). This was pronounced in adults without prior fractures (Additional file 2: Table S9). In multivariable-adjusted models, compared to participants with normal weight, overweight participants had a 5-year increase in the adjusted mean CFI of 0.01, those with obesity class I-II had an increase of 0.02, and those with morbid obesity had increases of 0.01 to 0.10 (Additional file 2: Figure S1, Figs 1, 2 and 3).

In addition to prior and incident fractures and obesity, some other predictors affected the progression of frailty: living alone (men: Additional file 2: Figure S1b and Fig. 2; *p* < 0.04), use of antiresorptives for 10 years (men: Additional file 2: Figure S1b and Fig. 2; *p* < 0.005), lower baseline BMD T-scores (Fig. 3; *p* = 0.02), and unemployment (Fig. 3; *p* = 0.03). Predictors that decelerated frailty were greater physical activity (women: Additional file 2: Figure S1a, Fig. 1 and both sexes: Fig. 3; p < 0.04), better HRQL (both sexes: Additional file 2: Figure S1, Figs 1-3; *p* < 0.0001), better cognitive function (women: Additional file 2: Figure S1a and Fig. 1; *p* < 0.05), university education (men: Additional file 2: Figure S1b; *p* = 0.04), and low alcohol consumption (women: Additional file 2: Figure S1a; p = 0.03).

Over 10 years, 3411 participants (44%) were lost to follow-up. Compared to participants who remained in the study, they were frailer (mean CFI: 0.18 ± 0.12), older, more often men, more often retired and living alone, more often underweight, less physically active, more often smokers, with prior low-trauma fractures and falls, with lower HRQL and cognitive scores (all *p*-values < 0.05, Additional file 2: Table S13). As expected, adults who died by year 10 had much greater CFI scores than those who dropped out (Additional file 2: Table S14). Our sensitivity analyses that addressed attrition or missing data bias corroborated the main findings (Additional file 2: Tables S15-S23), and also suggested a protective effect of non-smoking with the corresponding frailty 5-year reduction of 0.01 to 0.03 in the adjusted mean CFI (*p* < 0.01, Additional file 2: Tables S17-S23).

## Discussion

This study explored which predictors strongly affect the progression or deceleration of frailty over time in a population-based sample including over 7500 Canadian women and men aged 50 years and older. Two modifiable risk factors, incident low-trauma fractures and obesity, consistently increased frailty. Their effects on frailty progression were also clinically plausible because the rates of change were greater than a previously recognized minimal clinically important difference of 0.04 [16]. The long-term effects of low-trauma fractures were different between two sexes and depended on the history of fracture. In women who never had a low-trauma fracture, incident hip and clinical vertebral fractures had a similar impact on the progression of frailty; in contrast, an incident hip fracture was the only osteoporotic fracture associated with frailty progression in men. Prior low-trauma fracture intrinsically implies a different starting state [18]. Thus, individuals with prior fractures had more comorbidities and were frailer at baseline. In this subgroup, irrespective of the sex, an incident hip fracture significantly increased frailty over 10 years. Obesity consistently contributed to frailty progression in both women and men. Compared to normal weight participants (baseline BMI:25.0–29.9 kg/m^2^), the adjusted mean CFI increased by 0.01 per 5 years in overweight individuals (25.0–29.9 kg/m^2^) and was at least five times greater in pathologically obese individuals (BMI≥ 40.0 kg/m^2^). The impact was greater in adults who had no history of fractures and were less frail at baseline. Our analyses also showed that quality of life and physical activity protected against frailty; however, their impact might not be considered clinically meaningful because it is much smaller than the effect of fractures and obesity. Thus, greater HRQL scores suggesting better participants’ perceptions of physical and mental health were associated with decreases in frailty over time. Moreover, achieving a recommended weekly amount of physical activity of 1000 kilocals, which equals to approximately 30 min of walking per day [14, 15], significantly slowed down frailty over time in women without prior fractures and in adults with prior fractures.

Our findings have important public health implications and support hypotheses that frailty has a dynamic nature and may be attenuated through interventions aimed towards prevention and treatment of low-trauma fractures and obesity [4, 5, 7, 19]. Significant increases in the risks of hip and non-vertebral fractures in frail compared with robust populations were found in previous studies [8, 16, 20, 21]. Also, a significant increase of the Frailty Index score by 0.08 was found up to 2 years after a major osteoporotic fracture in frail elderly women with prior fractures (the mean baseline score: 0.24) [20]. Our study adds to this literature by showing that in women, incident clinical vertebral fractures may have a similar impact on the progression of frailty as incident hip fractures. Vertebral fractures were previously found to increase the risk of death [22], and to substantially affect individual’s quality of life [23]. Therefore, an early detection and treatment of vertebral fractures may be crucial for older women who are at a greater baseline risk of developing severe frailty than older men [24, 25].

Negative effects of obesity on frailty were suggested in other studies that showed a correlation between BMI and frailty, and increases in risks of fractures and falls in obese individuals [19, 26–28]. A relationship between obesity and frailty is complex and best explained through the phenomenon of sarcopenic obesity that represents a disproportional increase in fat mass as compared to the amount of muscle mass [21, 29–31]. This imbalance amplifies with aging, leading to poor muscle strength, poor muscle quality, and increased disability [4, 29, 31]. However, our study suggests that physical activity decelerates frailty even after controlling for the negative effects of fractures and obesity, which is in agreement with the findings of several systematic reviews suggesting a reduction of frailty with exercise interventions [4].

Frailty is a multidimensional concept including both psychosocial impairments and a physical function decline; consequently, a holistic approach for treating frailty has been suggested [7]. Our study findings support this recommendation: although the strongest predictors of frailty progression were related to physical frailty, factors related to a psychosocial construct such as HRQL, cognitive functioning, and living arrangement also represented important contributors. Current research has been mainly focused on examining causes, pharmacological and non-pharmacological treatments for sarcopenia [32, 33]. Future studies should examine if culturally tailored psychosocial interventions improve older adults’ well-being and their compliance to interventions aimed to decelerate physical frailty.

Our study has some limitations. CaMos is a population-based community dwelling study that did not include institutionalized seniors or those with cognitive impairments who are potentially the frailest of all. Also, non-linear changes in CFI, observed in some cohorts, indicate that although variability in a degree of frailty exists among individuals, any increase is conditioned on the starting state and any decline signifies survivor effects. Non-participation, survivor or attrition bias in epidemiologic studies of older people represents a common threat for the generalisability of study findings [34]. Despite this, our conclusions remained robust in sensitivity analyses.

## Conclusions

We showed that older adults with incident clinical vertebral fractures, hip fractures or obesity are at risk for more rapid progression of frailty. These individuals represent high-risk groups that should be considered for tailored frailty interventions. Future research should examine how nutrition, exercise and non-smoking interventions aimed at promoting healthy lifestyle for reducing frailty can be combined with psycho-social supports so that a short-term decrease in frailty progression transforms into a long-term reversal resulting in overall improvements of adults’ well-being and the optimal process of aging.

## Additional files

Additional file 1: Table S1. The CaMos Frailty Index. This document presents a table that describes 30 items included in the Camos Frailty Index. Source: Kennedy CC, Ioannidis G, Rockwood K, Thabane L, Adachi JD, Kirkland S, et al. A Frailty Index predicts 10-year fracture risk in adults age 25 years and older: results from the Canadian Multicentre Osteoporosis Study (CaMos). Osteoporos Int. 2014; 25:2825–32. Obtained with copyright permission (November 14,2017). (DOC 100 kb)

Additional file 2: Additional Results. This document presents additional results of the analysis. It contains 2 figures (Supplemental Figures S1a and S1b) and 23 tables (Supplemental Tables S1 to S23) (DOCX 281 kb)

## Abbreviations

BMD: Bone mineral density
BMI: Body mass index in kg/m^2^
CaMos: The Canadian Multicentre Osteoporosis Study
CFI: CaMos Frailty Index
CI: Confidence interval
HRQL: Health-related quality of life
METs: Metabolic equivalents
MMSE: MMSE by the Mini Mental State Examination
NHNV Fractures: Non-hip non-vertebral fractures
SD: Standard deviations
VF: Vertebral fractures
