# Supplementary material for: Osteoporotic fractures and obesity affect frailty progression: a longitudinal analysis of the Canadian multicentre osteoporosis study
Source: BMC Geriatr. 2018 Jan 5;18:4. doi: 10.1186/s12877-017-0692-0 (PMC5756402; doi:10.1186/s12877-017-0692-0)
Supplement: Supplementary file 2 — This document presents additional results of the analysis. It contains 2 figures (Supplemental Figures S1a and S1b) and 23 tables (Supplemental Tables S1 to S23) (DOCX 281 kb) [file 12877_2017_692_MOESM2_ESM.docx]

**Supplemental Files: Additional Results**

**FIGURES**

**Supplemental Figure S1a.** Changes in the Canadian Multicentre Osteoporosis (CaMos) Study Frailty Index (CFI) per a 5-year period in women aged 50+ years regardless of history of fracture: Significant predictors.

**Supplemental Figure S1b.** Changes in the Canadian Multicentre Osteoporosis Study (CaMos) Frailty Index (CFI) per a 5-year period in men aged 50+ years regardless of history of fracture: Significant predictors.

**TABLES**

**Supplemental Table S1.** Prevalent (baseline) and incident fractures in the study sample.

**Supplemental Table S2.** Changes in the CFI score over time in CaMos participants **with or without prior fracture**: Univariate GEE models including age, age*time interaction and incident fractures.

**Supplemental Table S3.** Changes in the CFI score over time in CaMos participants **without prior fracture**: Univariate GEE models including age, age*time interaction and incident fractures.

**Supplemental Table S4.** Changes in the CFI score over time in CaMos participants **with prior fracture**: Univariate GEE models including age, age*time interaction, sex and incident fractures.

**Supplemental Table S5.** Changes in the CFI score over time in CaMos participants **with or without prior fracture**: Univariate GEE models including age, age*time interaction and body mass index.

**Supplemental Table S6.** Changes in the CFI score over time in CaMos participants **without prior fracture**: Univariate GEE models including age, age*time interaction and body mass index.

**Supplemental Table S7.** Changes in the CFI score over time in CaMos participants **with prior fracture**: Univariate GEE models including age, age*time interaction, sex and body mass index.

**Supplemental Table S8.** Changes in the CFI score over time in CaMos participants **with or without prior fracture**: The effects of multiple low-trauma fractures.

**Supplemental Table S9.** Effects of incident low-trauma fractures, body mass index and other modifiable and non-modifiable factors on longitudinal changes in the CFI score in CaMos participants **without prior fracture.**

**Supplemental Table S10.** Changes in the CFI score over time in CaMos participants **without prior fracture**: The effects of multiple low-trauma fractures.

**Supplemental Table S11.**  Effects of incident low-trauma fractures, body mass index and other modifiable and non-modifiable factors on longitudinal changes in the CFI score in CaMos participants **with prior fracture.**

**Supplemental Table S12.** Change in the CFI score over time in CaMos participants **with prior fracture**: The effects of low-trauma multiple fractures.

**Supplemental Table S13.** Descriptive analysis: Loss to follow-up at year 10.

**Supplemental Table S14.** Differences in the CFI scores between CaMos participants who remained in the study, were lost-to follow-up or died by the end of year 10.

**Supplemental Table S15.** Missing data analysis: Longitudinal changes in the CFI scores in CaMos participants **with or without prior fracture.**

**Supplemental Table S16.** Missing data analysis: Longitudinal changes in the CFI scores in CaMos participants **without prior fracture.**

**Supplemental Table S17.** Missing data analysis: Longitudinal changes in the CFI scores in CaMos participants **with prior fracture.**

**Supplemental Table S18.** Worst Case Scenario 1 - imputations of the highest hip fracture-related CFI scores for CaMos participants who were lost-to-follow-up: Analyses in the sample **with or without prior fracture.**

**Supplemental Table S19.** Worst Case Scenario 1 - imputations of the highest hip fracture-related CFI scores for CaMos participants who were lost-to-follow-up: Analyses in the sample **without prior fracture.**

**Supplemental Table S20.** Worst Case Scenario 1 - imputations of the highest hip fracture-related CFI scores for CaMos participants who were lost-to-follow-up: Analyses in the sample **with prior fracture.**

**Supplemental Table S21.** Worst Case Scenario 2 - imputations of the upper theoretical CFI limit for CaMos participants who were lost-to-follow-up: Analyses in the sample **with or without prior fracture.**

**Supplemental Table S22.** Worst Case Scenario 2 - imputations of the upper theoretical CFI limit for CaMos participants who were lost-to-follow-up: Analyses in the sample **without prior fracture.**

**Supplemental Table S23.** Worst Case Scenario 2 - imputations of the upper theoretical CFI limit for CaMos participants who were lost-to-follow-up: Analyses in the sample **with prior fracture.**


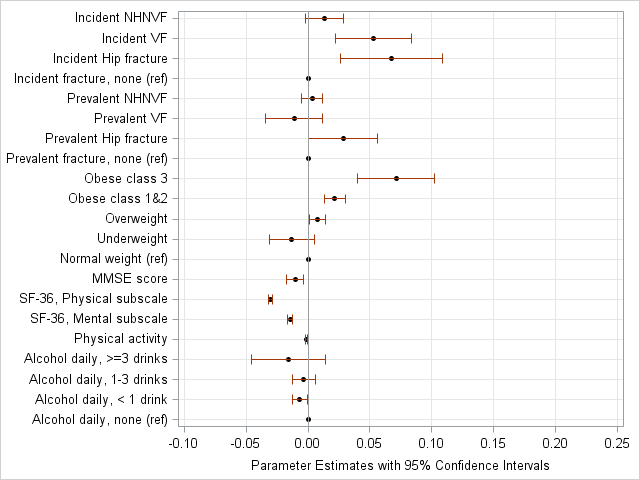


**Supplemental Figure S1a. Changes in the Canadian Multicentre Osteoporosis Study Frailty Index (CFI) per a 5-year period in women aged 50+ years regardless of history of fracture: Significant predictors**. Plots represent statistically significant risk factors shown in the multivariable-adjusted model (Table 3). Parameter estimates are regression coefficients that denote the mean change (increase: the value >0, and decrease: the value <0) in the CFI score per a 5-year period for one unit change/category in a predictor. NHNVF denotes non-hip-non-clinical vertebral fracture, VF denotes clinical vertebral fracture; ref denotes the reference group; MMSE denotes Mini Mental State Examination score and the value represent a decrease in frailty over 5 years per a 3.72-point change in the score. The parameter estimate for physical activity denotes a decrease in frailty per 1000 kilocals weekly; for SF-36 scores, decreases in frailty over 5 years are calculated per a 5-point change in the score.


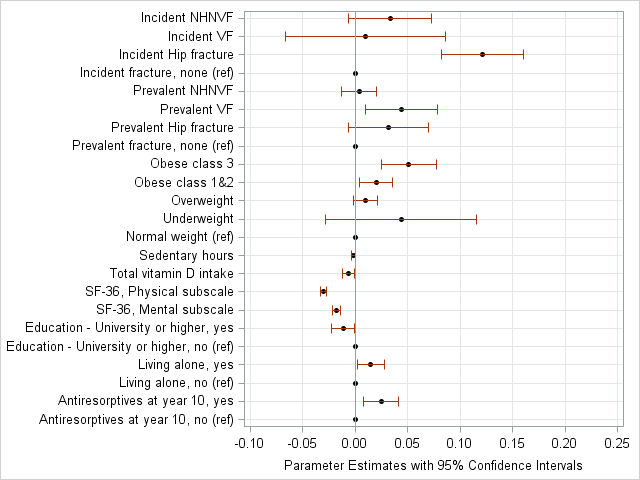


**Supplemental Figure S1b.** **Changes in the Canadian Multicentre Osteoporosis Study Frailty Index (CFI)** **per a 5-year period in men aged 50+ years regardless of history of fracture: Significant predictors**. Plots represent statistically significant risk factors shown in the multivariable-adjusted model (Table 3). Parameter estimates are regression coefficients that denote the mean change (increase: the value >0, and decrease: the value <0) in the CFI score per a 5-year period for one unit change/category in a predictor. NHNVF denotes non-hip-non-clinical vertebral fracture, VF denotes clinical vertebral fracture; ref denotes the reference group. The parameter estimate for a total vitamin D intake denotes a decrease in frailty over 5 years per 800 IU daily; for SF-36 scores, decreases in frailty over 5 years are calculated per a 5-point change in the score.

**Supplemental Table S1.** Prevalent (baseline) and incident fractures in the study sample.

|  | **Total** | **Women** | **Men** |
| --- | --- | --- | --- |
| **Prevalent Fractures** |  |  |  |
| Fracture*, by location, baseline, % (n/N):  Hip  Clinical vertebral  Non-hip-non-vertebral | 1.13 (88/7737)  0.85 (64/7737)  11.84 (918/7737) | 1.36 (76/5554)  0.99 (55/5554)  12.70 (706/5554) | 0.55 (12/2183)  0.41 (9/2183)  9.71 (212/2183) |
| Fracture*, by number, baseline, % (n/N):  Single  Multiple | 12.41 (960/7737)  1.42 (110/7737) | 13.38 (743/5554)  1.69 (94/5554) | 9.94 (217/2183)  0.73 (16/2183) |
| **Incident Fractures** |  |  |  |
| New low trauma fractures* year 1 to 5, % (n/N) | 7.36 (560/7606) | 8.69 (476/5475) | 3.94 (84/2131) |
| New low trauma fractures* year 6 to 10, % (n/N) | 8.12 (484/5961) | 9.55 (417/4365) | 4.20 (67/1596) |
| Fracture, by location, year 1 to 5, % (n/N):  Hip  Clinical vertebral  Non-hip-non-vertebral | 0.87 (66/7604)  0.97 (74/7604)  4.01 (305/7604) | 1.00 (55/5473)  1.24 (68/5473)  4.66 (255/5473) | 0.52 (11/2131)  0.28 (6/2131)  2.35 (50/2131) |
| Fracture, by location, year 6 to 10, % (n/N):  Hip  Clinical vertebral  Non-hip-non-vertebral | 1.64 (98/5959)  1.06 (63/5959)  4.03 (240/5959) | 1.72 (75/4363)  1.26 (55/4363)  4.81 (210/4363) | 1.44 (23/1596)  0.50 (8/1596)  1.88 (30/1596) |
| Fracture, by number, year 1 to 5, % (n/N):  Single  Multiple | 5.42 (412/7604)  0.43 (33/7604) | 6.36 (348/5473)  0.55 (30/5473) | 3.00 (64/2131)  0.14 (3/2131) |
| Fracture, by number, year 6 to 10, % (n/N):  Single  Multiple | 6.02 (359/5959)  0.70 (42/5959) | 6.99 (305/4363)  0.80 (35/4363) | 3.38 (54/1596)  0.44 (7/1596) |

***** excluding head, toe and finger fractures

**Supplemental Table S2.** Changes in the CFI score over time in CaMos participants **with or without prior fracture**: Univariate GEE models including age, age*time interaction and incident fractures.

|  | | | **Women** | | | | **Men** | | | |
| --- | --- | --- | --- | --- | --- | --- | --- | --- | --- | --- |
| **Parameter** | | | **Estimate** | **95% CI** | | **P-value** | **Estimate** | **95% CI** | | **P-value** |
| Intercept | | | 0.1159 | 0.1119 | 0.1198 | <.0001 | 0.0922 | 0.087 | 0.0975 | <.0001 |
| Time | 10 year |  | -0.0109 | -0.0147 | -0.007 | <.0001 | -0.0045 | -0.0101 | 0.0011 | 0.1125 |
|  | 5 year |  | 0.0181 | 0.0149 | 0.0213 | <.0001 | 0.0118 | 0.0071 | 0.0166 | <.0001 |
|  | Baseline (ref) |  | 0 |  |  |  | 0 |  |  |  |
| Age, years | >=80 |  | 0.1262 | 0.1154 | 0.1369 | <.0001 | 0.1014 | 0.085 | 0.1178 | <.0001 |
|  | 65-80 |  | 0.0482 | 0.0424 | 0.054 | <.0001 | 0.0389 | 0.031 | 0.0468 | <.0001 |
|  | 50-65 (ref) |  | 0 |  |  |  | 0 |  |  |  |
| Age*Time interaction | 10 year | >=80 | 0.028 | 0.0075 | 0.0485 | 0.0074 | 0.0403 | -0.0024 | 0.083 | 0.0642 |
|  |  | 65-80 | 0.0182 | 0.0122 | 0.0242 | <.0001 | 0.0186 | 0.0088 | 0.0283 | 0.0002 |
|  |  | 50-65 | 0 |  |  |  | 0 |  |  |  |
|  | 5 year | >=80 | 0.024 | 0.0117 | 0.0362 | 0.0001 | 0.0444 | 0.0218 | 0.067 | 0.0001 |
|  |  | 65-80 | 0.0179 | 0.0132 | 0.0227 | <.0001 | 0.0238 | 0.0161 | 0.0315 | <.0001 |
|  |  | 50-65 | 0 |  |  |  | 0 |  |  |  |
|  | Baseline (ref) | >=80 | 0 |  |  |  | 0 |  |  |  |
|  |  | 65-80 | 0 |  |  |  | 0 |  |  |  |
|  |  | 50-65 | 0 |  |  |  | 0 |  |  |  |
| Incident Fracture | NHNVF |  | 0.0111 | 0.0056 | 0.0166 | <.0001 | 0.0088 | -0.0012 | 0.0187 | 0.0832 |
|  | Clinical VF |  | 0.0419 | 0.0261 | 0.0578 | <.0001 | 0.0435 | 0.002 | 0.085 | 0.04 |
|  | Hip |  | 0.0488 | 0.0309 | 0.0666 | <.0001 | 0.0763 | 0.0403 | 0.1122 | <.0001 |
|  | None (ref) |  | 0 |  |  |  | 0 |  |  |  |

CFI= CaMos Frailty Index ; GEE= general estimating equations ; ref= the reference group;

NHNVF= non-hip non-clinical vertebral fractures; VF = vertebral fractures.

**Supplemental Table S3.** Changes in the CFI score over time in CaMos participants **without prior fracture**: Univariate GEE models including age, age*time interaction and incident fractures.

|  | | | **Women** | | | | **Men** | | | |
| --- | --- | --- | --- | --- | --- | --- | --- | --- | --- | --- |
| **Parameter** | | | **Estimate** | **95% CI** | | **P-value** | **Estimate** | **95% CI** | | **P-value** |
| Intercept | | | 0.1146 | 0.1104 | 0.1188 | <.0001 | 0.0919 | 0.0862 | 0.0975 | <.0001 |
| Time | 10 year |  | -0.0133 | -0.0175 | -0.0091 | <.0001 | -0.006 | -0.0121 | 0.0001 | 0.0552 |
|  | 5 year |  | 0.0154 | 0.012 | 0.0188 | <.0001 | 0.0094 | 0.0042 | 0.0146 | 0.0004 |
|  | Baseline (ref) |  | 0 |  |  |  | 0 |  |  |  |
| Age, years | >=80 |  | 0.1258 | 0.1132 | 0.1385 | <.0001 | 0.1035 | 0.085 | 0.1219 | <.0001 |
|  | 65-80 |  | 0.0468 | 0.0405 | 0.0532 | <.0001 | 0.0381 | 0.0294 | 0.0467 | <.0001 |
|  | 50-65 (ref) |  | 0 |  |  |  | 0 |  |  |  |
| Age*Time interraction | 10 year | >=80 | 0.0261 | 0.0007 | 0.0516 | 0.0439 | 0.0278 | -0.0212 | 0.0768 | 0.2661 |
|  |  | 65-80 | 0.0192 | 0.0126 | 0.0258 | <.0001 | 0.0192 | 0.0083 | 0.0301 | 0.0005 |
|  |  | 50-65 | 0 |  |  |  | 0 |  |  |  |
|  | 5 year | >=80 | 0.0321 | 0.0175 | 0.0466 | <.0001 | 0.0397 | 0.0155 | 0.0639 | 0.0013 |
|  |  | 65-80 | 0.0186 | 0.0134 | 0.0238 | <.0001 | 0.0226 | 0.0142 | 0.0311 | <.0001 |
|  |  | 50-65 | 0 |  |  |  | 0 |  |  |  |
|  | Baseline (ref) | >=80 | 0 |  |  |  | 0 |  |  |  |
|  |  | 65-80 | 0 |  |  |  | 0 |  |  |  |
|  |  | 50-65 | 0 |  |  |  | 0 |  |  |  |
| Incident Fracture | NHNVF |  | 0.0126 | 0.0024 | 0.0228 | 0.0156 | 0.0384 | 0.0132 | 0.0636 | 0.0028 |
|  | Clinical VF |  | 0.0525 | 0.029 | 0.076 | <.0001 | 0.0413 | -0.0154 | 0.0979 | 0.1533 |
|  | Hip |  | 0.0627 | 0.0274 | 0.098 | 0.0005 | 0.0802 | 0.0308 | 0.1296 | 0.0015 |
|  | None (ref) |  | 0 |  |  |  | 0 |  |  |  |

CFI= CaMos Frailty Index ; GEE= general estimating equations ; ref= the reference group;

NHNVF= non-hip non-clinical vertebral fractures; VF = vertebral fractures.

**Supplemental Table S4.** Changes in the CFI score over time in CaMos participants **with prior fracture**: Univariate GEE models including age, age*time interaction, sex and incident fractures.

| **Parameter** | | | **Estimate** | **95% CI** | | **P-value** |
| --- | --- | --- | --- | --- | --- | --- |
| Intercept | | | 0.0978 | 0.0868 | 0.1088 | <.0001 |
| Time | 10 year |  | -0.0067 | -0.0145 | 0.0011 | 0.0942 |
|  | 5 year |  | 0.0205 | 0.0136 | 0.0274 | <.0001 |
|  | Baseline (ref) |  | 0 |  |  |  |
| Age, years | >=80 |  | 0.1252 | 0.1069 | 0.1435 | <.0001 |
|  | 65-80 |  | 0.0521 | 0.0402 | 0.064 | <.0001 |
|  | 50-65 (ref) |  | 0 |  |  |  |
| Age*Time interaction | 10 year | >=80 | 0.0193 | -0.0144 | 0.0529 | 0.2619 |
|  |  | 65-80 | 0.0059 | -0.0059 | 0.0177 | 0.3245 |
|  |  | 50-65 | 0 |  |  |  |
|  | 5 year | >=80 | 0.0019 | -0.0203 | 0.024 | 0.87 |
|  |  | 65-80 | 0.0111 | 0.0016 | 0.0207 | 0.0228 |
|  |  | 50-65 | 0 |  |  |  |
|  | Baseline (ref) | >=80 | 0 |  |  |  |
|  |  | 65-80 | 0 |  |  |  |
|  |  | 50-65 | 0 |  |  |  |
| Female |  |  | 0.0337 | 0.0227 | 0.0447 | <.0001 |
| Incident Fracture | NHNVF |  | 0.0112 | -0.0065 | 0.0288 | 0.2148 |
|  | Clinical VF |  | 0.0387 | 0.004 | 0.0734 | 0.0288 |
|  | Hip |  | 0.0678 | 0.0327 | 0.1028 | 0.0002 |
|  | None (ref) |  | 0 |  |  |  |

CFI= CaMos Frailty Index ; GEE= general estimating equations ; ref= the reference group;

NHNVF= non-hip non-clinical vertebral fractures; VF = vertebral fractures.

**Supplemental Table S5.** Changes in the CFI score over time in CaMos participants **with or without prior fracture**: Univariate GEE models including age, age*time interaction and body mass index.

|  | | | **Women** | | | | **Men** | | | |
| --- | --- | --- | --- | --- | --- | --- | --- | --- | --- | --- |
| **Parameter** | | | **Estimate** | **95% CI** | | **P-value** | **Estimate** | **95% CI** | | **P-value** |
| Intercept | | | 0.095 | 0.0902 | 0.0998 | <.0001 | 0.0836 | 0.0759 | 0.0912 | <.0001 |
| Time | 10 year |  | -0.0107 | -0.0145 | -0.0069 | <.0001 | -0.0047 | -0.0101 | 0.0008 | 0.0963 |
|  | 5 year |  | 0.0174 | 0.0142 | 0.0206 | <.0001 | 0.0114 | 0.0068 | 0.0161 | <.0001 |
|  | Baseline (ref) |  | 0 |  |  |  | 0 |  |  |  |
| Age, years | >=80 |  | 0.1326 | 0.1217 | 0.1435 | <.0001 | 0.1068 | 0.0895 | 0.1241 | <.0001 |
|  | 65-80 |  | 0.0494 | 0.0438 | 0.055 | <.0001 | 0.0397 | 0.0318 | 0.0476 | <.0001 |
|  | 50-65 (ref) |  | 0 | 0 | 0 | . | 0 |  |  |  |
| Age*Time interaction | 10 year | >=80 | 0.0325 | 0.0114 | 0.0537 | 0.0026 | 0.0418 | -0.003 | 0.0865 | 0.0673 |
|  |  | 65-80 | 0.0181 | 0.0121 | 0.0241 | <.0001 | 0.019 | 0.0092 | 0.0289 | 0.0002 |
|  |  | 50-65 | 0 | 0 | 0 | . | 0 | 0 | 0 | . |
|  | 5 year | >=80 | 0.0268 | 0.0141 | 0.0395 | <.0001 | 0.0463 | 0.0226 | 0.0699 | 0.0001 |
|  |  | 65-80 | 0.0173 | 0.0125 | 0.022 | <.0001 | 0.0247 | 0.0169 | 0.0324 | <.0001 |
|  |  | 50-65 | 0 |  |  |  | 0 |  |  |  |
|  | Baseline (ref) | >=80 | 0 |  |  |  | 0 |  |  |  |
|  |  | 65-80 | 0 |  |  |  | 0 |  |  |  |
|  |  | 50-65 | 0 |  |  |  | 0 |  |  |  |
| BMI, kg/m2 | >=40 |  | 0.1376 | 0.1132 | 0.1621 | <.0001 | 0.1281 | 0.0774 | 0.1789 | <.0001 |
|  | 30-40 |  | 0.048 | 0.0411 | 0.0548 | <.0001 | 0.0244 | 0.0139 | 0.0348 | <.0001 |
|  | 25-30 |  | 0.0172 | 0.0117 | 0.0227 | <.0001 | 0.0057 | -0.0026 | 0.0139 | 0.1774 |
|  | <18.5 |  | 0.0283 | 0.0054 | 0.0513 | 0.0157 | 0.038 | -0.0083 | 0.0844 | 0.108 |
|  | 18.5-25 (ref) |  | 0 |  |  |  | 0 |  |  |  |

CFI= CaMos Frailty Index ; GEE= general estimating equations ; ref= the reference group; BMI= body mass index.

**Supplemental** **Table S6.** Changes in the CFI score over time in CaMos participants **without prior fracture**: Univariate GEE models including age, age*time interaction and body mass index.

|  | | | **Women** | | | | **Men** | | | |
| --- | --- | --- | --- | --- | --- | --- | --- | --- | --- | --- |
| **Parameter** | | | **Estimate** | **95% CI** | | **P-value** | **Estimate** | **95% CI** | | **P-value** |
| Intercept | | | 0.0918 | 0.0868 | 0.0969 | <.0001 | 0.0808 | 0.0727 | 0.0889 | <.0001 |
| Time | 10 year |  | -0.0117 | -0.0159 | -0.0076 | <.0001 | -0.0049 | -0.011 | 0.0012 | 0.1171 |
|  | 5 year |  | 0.0162 | 0.0128 | 0.0196 | <.0001 | 0.0104 | 0.0051 | 0.0156 | 0.0001 |
|  | Baseline (ref) |  | 0 |  |  |  | 0 |  |  |  |
| Age, years | >=80 |  | 0.1321 | 0.1192 | 0.1449 | <.0001 | 0.1079 | 0.0885 | 0.1273 | <.0001 |
|  | 65-80 |  | 0.0468 | 0.0407 | 0.0529 | <.0001 | 0.0378 | 0.0292 | 0.0464 | <.0001 |
|  | 50-65 |  | 0 |  |  |  | 0 |  |  |  |
| Age*Time interaction | 10 year | >=80 | 0.0308 | 0.0043 | 0.0574 | 0.0228 | 0.0295 | -0.0228 | 0.0817 | 0.2691 |
|  |  | 65-80 | 0.0203 | 0.0137 | 0.0269 | <.0001 | 0.0208 | 0.0099 | 0.0318 | 0.0002 |
|  |  | 50-65 | 0 |  |  |  | 0 |  |  |  |
|  | 5 year | >=80 | 0.0372 | 0.0222 | 0.0522 | <.0001 | 0.0425 | 0.0174 | 0.0676 | 0.0009 |
|  |  | 65-80 | 0.0193 | 0.0141 | 0.0245 | <.0001 | 0.0246 | 0.0161 | 0.0331 | <.0001 |
|  |  | 50-65 | 0 |  |  |  | 0 |  |  |  |
|  | Baseline (ref) | >=80 | 0 |  |  |  | 0 |  |  |  |
|  |  | 65-80 | 0 |  |  |  | 0 |  |  |  |
|  |  | 50-65 | 0 |  |  |  | 0 |  |  |  |
| BMI, kg/m2 | >=40 |  | 0.1444 | 0.1174 | 0.1714 | <.0001 | 0.1336 | 0.0783 | 0.1888 | <.0001 |
|  | 30-40 |  | 0.0465 | 0.0389 | 0.054 | <.0001 | 0.025 | 0.014 | 0.0361 | <.0001 |
|  | 25-30 |  | 0.0204 | 0.0144 | 0.0264 | <.0001 | 0.0083 | -0.0005 | 0.017 | 0.0632 |
|  | <18.5 |  | 0.0041 | -0.022 | 0.0302 | 0.7588 | 0.0322 | -0.0167 | 0.0811 | 0.1969 |
|  | 18.5-25 (ref) |  | 0 |  |  |  | 0 |  |  |  |

CFI= CaMos Frailty Index ; GEE= general estimating equations ; ref= the reference group; BMI= body mass index.

**Supplemental** **Table S7.** Changes in the CFI score over time in CaMos participants **with prior fracture**: Univariate GEE models including age, age*time interaction, sex and body mass index.

| **Parameter** | | | **Estimate** | **95% CI** | | **P-value** |
| --- | --- | --- | --- | --- | --- | --- |
| Intercept | | | 0.0881 | 0.0744 | 0.1018 | <.0001 |
| Time | 10 year |  | -0.0049 | -0.0126 | 0.0028 | 0.2141 |
|  | 5 year |  | 0.0212 | 0.0143 | 0.0281 | <.0001 |
|  | Baseline (ref) |  | 0 |  |  |  |
| Age, years | >=80 |  | 0.1208 | 0.1022 | 0.1393 | <.0001 |
|  | 65-80 |  | 0.0529 | 0.0413 | 0.0646 | <.0001 |
|  | 50-65 (ref) |  | 0 |  |  |  |
| Age*Time interaction | 10 year | >=80 | 0.0372 | 0.0044 | 0.07 | 0.0262 |
|  |  | 65-80 | 0.009 | -0.0028 | 0.0209 | 0.134 |
|  |  | 50-65 | 0 | 0 | 0 | . |
|  | 5 year | >=80 | 0.0115 | -0.0114 | 0.0343 | 0.3255 |
|  |  | 65-80 | 0.0127 | 0.003 | 0.0224 | 0.0106 |
|  |  | 50-65 | 0 |  |  |  |
|  | Baseline (ref) | >=80 | 0 |  |  |  |
|  |  | 65-80 | 0 |  |  |  |
|  |  | 50-65 | 0 |  |  |  |
| Female |  |  | 0.0263 | 0.0152 | 0.0374 | <.0001 |
| BMI, kg/m2 | >=40 |  | 0.109 | 0.0573 | 0.1606 | <.0001 |
|  | 30-40 |  | 0.046 | 0.0316 | 0.0604 | <.0001 |
|  | 25-30 |  | 0 | -0.0115 | 0.0115 | 0.9997 |
|  | <18.5 |  | 0.0708 | 0.0333 | 0.1083 | 0.0002 |
|  | 18.5-25 (ref) |  | 0 |  |  |  |

CFI= CaMos Frailty Index ; GEE= general estimating equations ; ref= the reference group; BMI= body mass index.

**Supplemental Table S8.** Changes in the CFI score over time in CaMos participants **with or without prior fracture**: The effects of multiple low-trauma fractures.

| **Parameter** | | | **Women** | | | | **Men** | | | |
| --- | --- | --- | --- | --- | --- | --- | --- | --- | --- | --- |
|  |  |  | **Estimate** | **95% CI** | | **P-value** | Estimate | **95% CI** | | **P-value** |
| Time | 10 year | | -0.0954 | -0.2006 | 0.0097 | 0.0753 | -0.0083 | -0.0083 | -0.0083 | - |
|  | 5 year | | -0.0219 | -0.1231 | 0.0794 | 0.6719 | -0.05 | -0.05 | -0.05 | - |
|  | Baseline (reference) | | 0.000 |  |  |  | 0.000 |  |  |  |
| Age, years | >=80 | | -0.0043 | -0.0726 | 0.0639 | 0.9013 | 0.0638 | 0.0299 | 0.0976 | 0.0002 |
|  | 65-80 | | -0.0301 | -0.097 | 0.0368 | 0.3774 | 0.0599 | 0.0381 | 0.0817 | <.0001 |
|  | 50-65 (reference) | | 0.000 |  |  |  | 0.000 |  |  |  |
| Time*Age interaction | 10 year | >=80 | **0.1153** | 0.0068 | 0.2239 | **0.0372** | **0.0777** | 0.0378 | 0.1176 | **0.0001** |
|  |  | 65-80 | 0.1049 | -0.0004 | 0.2102 | 0.0509 | **0.0288** | 0.0201 | 0.0374 | **<.0001** |
|  |  | 50-65 | 0 |  |  |  | 0 |  |  |  |
|  | 5 year | >=80 | 0.0425 | -0.0616 | 0.1467 | 0.4233 | **0.0995** | 0.0603 | 0.1387 | **<.0001** |
|  |  | 65-80 | 0.0488 | -0.0526 | 0.1501 | 0.3454 | **0.0819** | 0.0746 | 0.0892 | **<.0001** |
|  |  | 50-65 | 0.000 |  |  |  | 0.000 |  |  |  |
|  | Baseline (reference) | >=80 | 0.000 |  |  |  | 0.000 |  |  |  |
|  |  | 65-80 | 0.000 |  |  |  | 0.000 |  |  |  |
|  |  | 50-65 | 0.000 |  |  |  | 0.000 |  |  |  |
| Prevalent Fracture | Multiple | | 0.017 | -0.0043 | 0.0382 | 0.1172 | **0.0336** | 0.0091 | 0.0581 | **0.0072** |
|  | Single | | 0.0023 | -0.0061 | 0.0106 | 0.5963 | 0.0051 | -0.0118 | 0.022 | 0.5517 |
|  | None (reference) | | 0.000 |  |  |  |  |  |  |  |
| Incident Fracture | Multiple | | **0.0611** | 0.0124 | 0.1098 | **0.0139** | 0.0134 | -0.0568 | 0.0837 | 0.7077 |
|  | Single | | **0.0258** | 0.0115 | 0.0401 | **0.0004** | **0.0519** | 0.0187 | 0.0852 | **0.0022** |
|  | None (reference) | | 0.000 |  |  |  |  |  |  |  |
| Caucasian, yes | | | 0.0026 | -0.0116 | 0.0168 | 0.7187 | -0.0056 | -0.0289 | 0.0177 | 0.6396 |
| BMI, kg/m2 | >=40 | | **0.071** | 0.0403 | 0.1018 | **<.0001** | **0.0525** | 0.0258 | 0.0792 | **0.0001** |
|  | 30-40 | | **0.0214** | 0.0129 | 0.03 | **<.0001** | **0.0196** | 0.004 | 0.0351 | **0.0137** |
|  | 25-30 | | **0.0078** | 0.0014 | 0.0141 | **0.0161** | 0.01 | -0.0017 | 0.0218 | 0.0949 |
|  | <18.5 | | -0.0098 | -0.0288 | 0.0091 | 0.3092 | 0.0622 | -0.0288 | 0.1531 | 0.1803 |
|  | 18.5-25 (reference) | | 0.000 |  |  |  | 0.000 |  |  |  |
| Physical activity (kilocal/week, per 1000) | | | **-0.0014** | -0.0024 | -0.0004 | **0.0055** | -0.0002 | -0.0011 | 0.0007 | 0.7081 |
| Sedentary lifestyle, hours/day | | | 0.001 | -0.0001 | 0.0021 | 0.0834 | -0.0018 | -0.0036 | 0.0001 | 0.0583 |
| Total calcium intake (per 1200 mg/day) | | | 0.0032 | -0.0026 | 0.0091 | 0.2809 | 0.0012 | -0.0094 | 0.0117 | 0.8306 |
| Total vitamin D intake (per 800 IU /day) | | | -0.0002 | -0.0012 | 0.0008 | 0.6897 | -0.006 | -0.0122 | 0.0002 | 0.0572 |
| MMSE score (per 3.72 point change) | | | **-0.011** | -0.0181 | -0.0039 | **0.0023** | -0.0037 | -0.0165 | 0.0091 | 0.5705 |
| SF-36 - Physical subscale score (per 5 units change) | | | **-0.0306** | -0.0322 | -0.029 | **<.0001** | **-0.0299** | -0.0329 | -0.0269 | **<.0001** |
| SF-36 - Mental subscale score (per 5 units change) | | | **-0.0146** | -0.0165 | -0.0128 | **<.0001** | **-0.0182** | -0.0221 | -0.0144 | **<.0001** |
| Education: University or higher (yes) | | | -0.0028 | -0.0099 | 0.0042 | 0.4293 | -0.0101 | -0.0211 | 0.0009 | 0.0727 |
| Smoking | Never | | -0.0023 | -0.0128 | 0.0081 | 0.6588 | -0.0184 | -0.0412 | 0.0044 | 0.1141 |
|  | Past | | 0.0106 | 0 | 0.0212 | 0.0491 | -0.0065 | -0.0283 | 0.0153 | 0.5612 |
|  | Current (reference) | | 0.000 |  |  |  | 0.000 |  |  |  |
| Alcohol, drinks per day | >=3 | | -0.012 | -0.0443 | 0.0202 | 0.4642 | -0.0164 | -0.0377 | 0.0049 | 0.1318 |
|  | 1 to 3 | | -0.0036 | -0.013 | 0.0059 | 0.4623 | -0.0012 | -0.0155 | 0.0131 | 0.8685 |
|  | >0 to <1 | | **-0.0067** | -0.0128 | -0.0005 | **0.0333** | 0.0074 | -0.005 | 0.0199 | 0.2395 |
|  | None (reference) | | 0.000 |  |  |  | 0.000 |  |  |  |
| Employment | Unemployed | | 0.0002 | -0.0312 | 0.0316 | 0.9924 | -0.0156 | -0.0558 | 0.0246 | 0.4459 |
|  | Retired | | -0.0024 | -0.0187 | 0.0138 | 0.7691 | -0.0076 | -0.0221 | 0.0068 | 0.2997 |
|  | Homemaker | | 0.0028 | -0.0139 | 0.0195 | 0.7437 | - |  |  |  |
|  | Employed (reference) | | 0.000 |  |  |  | 0.000 |  |  |  |
| Living with a partner, no | | | 0.0027 | -0.0031 | 0.0085 | 0.354 | **0.0154** | 0.0028 | 0.028 | **0.0164** |
| BMD T-score at baseline | | | 0.0019 | -0.0016 | 0.0054 | 0.2849 | -0.0032 | -0.0092 | 0.0028 | 0.2923 |
| Antiresorptive treatment at baseline, yes | | | 0.0008 | -0.0064 | 0.008 | 0.8223 | -0.049 | -0.1007 | 0.0026 | 0.0629 |
| Antiresorptive treatment at year 10, yes | | | 0.004 | -0.0022 | 0.0103 | 0.2039 | **0.0266** | 0.0098 | 0.0434 | **0.0019** |
| Falls in past month , yes | | | 0.0024 | -0.0099 | 0.0148 | 0.6976 | 0.0161 | -0.0086 | 0.0407 | 0.2009 |
| Ever confined to bed (immobilized), yes | | | -0.0009 | -0.0104 | 0.0086 | 0.8528 | 0.0083 | -0.0052 | 0.0218 | 0.2275 |
| Ever lost more than 10 pounds, yes | | | 0.0046 | -0.0012 | 0.0103 | 0.1187 | 0.0049 | -0.005 | 0.0148 | 0.3314 |

CFI= CaMos Frailty Index; BMI= body mass index; MMSE = Mini Mental State Examination; BMD – bone mineral density. **Supplemental Table S9.** Effects of incident low-trauma fractures, body mass index and other modifiable and non-modifiable factors on longitudinal changes in the CFI score in CaMos participants **without prior fracture.**

| **Parameter** | | | **Women** | | | | **Men** | | | |
| --- | --- | --- | --- | --- | --- | --- | --- | --- | --- | --- |
|  |  |  | **Estimate** | **95% CI** | | **P-value** | **Estimate** | **95% CI** | | **P-value** |
| Time | 10 year | | -0.057 | -0.168 | 0.053 | 0.310 | -0.008 | -0.008 | -0.008 | <.0001 |
|  | 5 year | | 0.029 | -0.040 | 0.098 | 0.403 | -0.050 | -0.050 | -0.050 | <.0001 |
|  | Baseline (reference) | | 0.000 |  |  |  | 0.000 |  |  |  |
| Age, years | >=80 | | 0.028 | -0.025 | 0.080 | 0.303 | 0.055 | 0.018 | 0.092 | 0.003 |
|  | 65-80 | | 0.001 | -0.048 | 0.050 | 0.966 | 0.056 | 0.031 | 0.080 | <.0001 |
|  | 50-65 (reference) | | 0.000 |  |  |  | 0.000 |  |  |  |
| Time*Age | 10 year | >=80 | 0.068 | -0.047 | 0.183 | 0.249 | **0.084** | 0.038 | 0.130 | **0.000** |
|  |  | 65-80 | 0.068 | -0.043 | 0.178 | 0.231 | **0.028** | 0.019 | 0.038 | **<.0001** |
|  |  | 50-65 | 0.000 |  |  |  | 0.000 |  |  |  |
|  | 5 year | >=80 | -0.002 | -0.077 | 0.073 | 0.957 | **0.114** | 0.071 | 0.156 | **<.0001** |
|  |  | 65-80 | -0.002 | -0.071 | 0.068 | 0.965 | **0.081** | 0.073 | 0.089 | **<.0001** |
|  |  | 50-65 | 0.000 |  |  |  | 0.000 |  |  |  |
|  | Baseline (ref) | >=80 | 0.000 |  |  |  | 0.000 |  |  |  |
|  |  | 65-80 | 0.000 |  |  |  | 0.000 |  |  |  |
|  |  | 50-65 | 0.000 |  |  |  | 0.000 |  |  |  |
| Incident Fracture | NHNVF | | **0.019** | 0.003 | 0.035 | **0.022** | **0.050** | 0.004 | 0.096 | **0.035** |
|  | clinical VF | | **0.060** | 0.024 | 0.097 | **0.001** | -0.022 | -0.053 | 0.009 | 0.155 |
|  | Hip | | **0.069** | 0.012 | 0.127 | 0.017 | **0.110** | 0.060 | 0.160 | **<.0001** |
|  | None (reference) | | 0.000 |  |  |  | 0.000 |  |  |  |
| Caucasian , yes | | | 0.002 | -0.013 | 0.016 | 0.810 | -0.006 | -0.032 | 0.020 | 0.638 |
| BMI, kg/m2 | >=40 | | **0.101** | 0.070 | 0.131 | **<.0001** | **0.055** | 0.026 | 0.084 | **0.000** |
|  | 30-40 | | **0.023** | 0.014 | 0.033 | **<.0001** | **0.023** | 0.006 | 0.040 | **0.007** |
|  | 25-30 | | **0.008** | 0.001 | 0.016 | **0.026** | **0.013** | 0.001 | 0.026 | **0.037** |
|  | <18.5 | | -0.031 | -0.047 | -0.015 | 0.000 | 0.038 | -0.037 | 0.112 | 0.320 |
|  | 18.5-25 (reference) | | 0.000 |  |  |  | 0.000 |  |  |  |
| Physical activity (kilocal/week, per 1000) | | | **-0.001** | -0.002 | 0.000 | **0.036** | 0.000 | -0.001 | 0.001 | 0.983 |
| Sedentary lifestyle, hours/day | | | 0.001 | 0.000 | 0.002 | 0.172 | -0.001 | -0.003 | 0.001 | 0.246 |
| Total calcium intake (per 1200 mg/day) | | | 0.001 | -0.006 | 0.008 | 0.820 | 0.002 | -0.010 | 0.014 | 0.716 |
| Total vitamin D intake (per 800 IU /day) | | | -0.001 | -0.002 | 0.001 | 0.408 | -0.008 | -0.016 | 0.000 | 0.050 |
| MMSE score (per 3.72 point change) | | | **-0.008** | -0.017 | 0.000 | **0.049** | -0.006 | -0.020 | 0.009 | 0.454 |
| SF-36 - Physical subscale (per 5 unit change) | | | **-0.030** | -0.032 | -0.029 | **<.0001** | **-0.030** | -0.034 | -0.027 | **<.0001** |
| SF-36 - Mental subscale (per 5 unit change) | | | **-0.015** | -0.017 | -0.013 | **<.0001** | **-0.018** | -0.022 | -0.013 | **<.0001** |
| Education: University or higher (yes) | | | -0.002 | -0.011 | 0.006 | 0.594 | -0.011 | -0.023 | 0.001 | 0.070 |
| Smoking | Never | | 0.001 | -0.012 | 0.013 | 0.930 | -0.014 | -0.036 | 0.008 | 0.204 |
|  | Past | | 0.013 | 0.000 | 0.025 | 0.057 | -0.003 | -0.023 | 0.018 | 0.815 |
|  | Current (reference) | | 0.000 |  |  |  | 0.000 |  |  |  |
| Alcohol, drinks/day | >=3 | | -0.015 | -0.052 | 0.023 | 0.451 | -0.015 | -0.038 | 0.008 | 0.208 |
|  | 1 to 3 | | -0.004 | -0.015 | 0.007 | 0.514 | -0.002 | -0.019 | 0.014 | 0.772 |
|  | >0 to <1 | | -0.006 | -0.013 | 0.001 | 0.093 | 0.010 | -0.004 | 0.024 | 0.152 |
|  | None (reference) | | 0.000 |  |  |  | 0.000 |  |  |  |
| Employment | Unemployed | | -0.002 | -0.037 | 0.032 | 0.897 | -0.018 | -0.060 | 0.024 | 0.409 |
|  | Retired | | -0.004 | -0.024 | 0.015 | 0.668 | -0.007 | -0.023 | 0.009 | 0.396 |
|  | Homemaker | | 0.000 | -0.020 | 0.020 | 0.999 |  |  |  |  |
|  | Employed (reference) | | 0.000 |  |  |  | 0.000 |  |  |  |
| Living with a partner, no | | | 0.006 | -0.001 | 0.012 | 0.109 | **0.013** | 0.001 | 0.026 | **0.040** |
| BMD T-score at baseline | | | 0.001 | -0.003 | 0.005 | 0.760 | -0.006 | -0.013 | 0.000 | 0.067 |
| Antiresorptive treatment at baseline, yes | | | 0.002 | -0.007 | 0.010 | 0.671 | -0.041 | -0.099 | 0.017 | 0.163 |
| Antiresorptive treatment at year 10, yes | | | 0.004 | -0.003 | 0.011 | 0.265 | **0.030** | 0.011 | 0.048 | **0.002** |
| Falls in past month, yes | | | 0.005 | -0.009 | 0.018 | 0.515 | 0.015 | -0.016 | 0.046 | 0.347 |
| Ever confined to bed (immobilized), yes | | | 0.010 | -0.002 | 0.023 | 0.110 | 0.011 | -0.005 | 0.026 | 0.168 |
| Ever lost more than 10 pounds, yes | | | 0.003 | -0.004 | 0.009 | 0.416 | 0.011 | 0.000 | 0.022 | 0.056 |

CFI= CaMos Frailty Index; NHNVF= non-hip non-clinical vertebral fractures; VF = vertebral fractures. BMI= body mass index; MMSE = Mini Mental State Examination; BMD – bone mineral density.

**Supplemental Table S10.** Changes in the CFI score over time in CaMos participants **without prior fracture**: The effects of multiple low-trauma fractures.

| **Parameter** | | | **Women** | | | | **Men** | | | |
| --- | --- | --- | --- | --- | --- | --- | --- | --- | --- | --- |
|  |  |  | **Estimate** | **95% CI** | | **P-value** | **Estimate** | **95% CI** | | **P-value** |
| Time | 10 year |  | -0.0572 | -0.1676 | 0.0531 | 0.3095 | -0.0083 | -0.0083 | -0.0083 | <.0001 |
|  | 5 year |  | 0.0294 | -0.0395 | 0.0984 | 0.4025 | -0.05 | -0.05 | -0.05 | <.0001 |
|  | Baseline (reference) |  | 0.000 |  |  |  | 0.000 |  |  |  |
| Age, years | >=80 |  | 0.0276 | -0.0243 | 0.0796 | 0.2971 | 0.0554 | 0.0183 | 0.0924 | 0.0034 |
|  | 65-80 |  | 0.0013 | -0.0476 | 0.0502 | 0.9582 | 0.0558 | 0.0311 | 0.0804 | <.0001 |
|  | 50-65 (reference) |  | 0.000 |  |  |  | 0.000 |  |  |  |
| Time*Age | 10 year | >=80 | 0.0686 | -0.0465 | 0.1837 | 0.2426 | **0.0842** | 0.0379 | 0.1304 | **0.0004** |
|  |  | 65-80 | 0.0676 | -0.0429 | 0.1781 | 0.2308 | **0.0283** | 0.0186 | 0.0381 | **<.0001** |
|  |  | 50-65 | 0.000 |  |  |  | 0.000 |  |  |  |
|  | 5 year | >=80 | -0.0014 | -0.077 | 0.0742 | 0.9715 | **0.1135** | 0.0708 | 0.1562 | **<.0001** |
|  |  | 65-80 | -0.0017 | -0.0708 | 0.0675 | 0.9626 | **0.0807** | 0.0728 | 0.0886 | **<.0001** |
|  |  | 50-65 | 0.000 |  |  |  | 0.000 |  |  |  |
|  | Baseline (reference) | >=80 | 0.000 |  |  |  | 0.000 |  |  |  |
|  |  | 65-80 | 0.000 |  |  |  | 0.000 |  |  |  |
|  |  | 50-65 | 0.000 |  |  |  | 0.000 |  |  |  |
| Incident Fracture | Multiple | | 0.0484 | -0.0175 | 0.1144 | 0.1502 | 0.0145 | -0.0579 | 0.0869 | 0.6944 |
|  | Single | | **0.0321** | 0.0159 | 0.0482 | **<.0001** | **0.0595** | 0.0207 | 0.0982 | **0.0026** |
|  | None (reference) | | 0.000 |  |  |  |  |  |  |  |
| Caucasian, yes | | | 0.0019 | -0.0124 | 0.0162 | 0.7931 | -0.0057 | -0.0317 | 0.0204 | 0.6697 |
| BMI, kg/m2 | >=40 | | **0.1004** | 0.0699 | 0.131 | **<.0001** | **0.0549** | 0.0254 | 0.0843 | **0.0003** |
|  | 30-40 | | **0.0227** | 0.0131 | 0.0324 | **<.0001** | **0.022** | 0.0052 | 0.0388 | **0.0102** |
|  | 25-30 | | **0.0083** | 0.0009 | 0.0156 | **0.0271** | **0.013** | 0.0007 | 0.0254 | **0.0389** |
|  | <18.5 | | **-0.0302** | -0.047 | -0.0135 | **0.0004** | 0.0541 | -0.0387 | 0.1468 | 0.2535 |
|  | 18.5-25 (reference) | | 0.000 |  |  |  | 0.000 |  |  |  |
| Physical activity (kilocal/week, per 1000) | | | **-0.0012** | -0.0023 | -0.0001 | **0.0349** | 0.0001 | -0.0013 | 0.0014 | 0.9394 |
| Sedentary lifestyle, hours/day | | | 0.0009 | -0.0004 | 0.0021 | 0.1865 | -0.0012 | -0.0033 | 0.001 | 0.2833 |
| Total calcium intake (per 1200 mg/day) | | | 0.0009 | -0.0059 | 0.0077 | 0.7898 | 0.0021 | -0.0099 | 0.0141 | 0.733 |
| Total vitamin D intake (per 800 IU /day) | | | -0.0006 | -0.0019 | 0.0007 | 0.3939 | -0.0084 | -0.0168 | 0 | 0.0507 |
| MMSE score (per 3.72 point change) | | | **-0.0085** | -0.0169 | -0.0001 | **0.0478** | -0.0053 | -0.02 | 0.0095 | 0.4841 |
| SF-36 - Physical subscale (per 5 unit change) | | | **-0.0304** | -0.0323 | -0.0285 | **<.0001** | **-0.0302** | -0.0336 | -0.0269 | **<.0001** |
| SF-36 - Mental subscale (per 5 unit change) | | | **-0.0147** | -0.0169 | -0.0125 | **<.0001** | **-0.0178** | -0.022 | -0.0135 | **<.0001** |
| Education: University or higher | | | -0.0021 | -0.0103 | 0.0062 | 0.621 | -0.0106 | -0.0226 | 0.0013 | 0.0812 |
| Smoking | Never | | 0.0003 | -0.0124 | 0.0129 | 0.9644 | -0.0149 | -0.0367 | 0.007 | 0.1822 |
|  | Past | | 0.0125 | -0.0003 | 0.0253 | 0.0559 | -0.0032 | -0.0241 | 0.0177 | 0.7634 |
|  | Current (reference) | | 0.000 |  |  |  | 0.000 |  |  |  |
| Alcohol, drink/day | >=3 | | -0.0147 | -0.0524 | 0.0231 | 0.446 | -0.0154 | -0.0385 | 0.0078 | 0.1933 |
|  | 1 to 3 | | -0.0038 | -0.0148 | 0.0072 | 0.4998 | -0.0022 | -0.0186 | 0.0142 | 0.7929 |
|  | >0 to <1 | | -0.0061 | -0.0131 | 0.001 | 0.0926 | 0.0096 | -0.0042 | 0.0235 | 0.1726 |
|  | None (reference) | | 0.000 |  |  |  | 0.000 |  |  |  |
| Employment | Unemployed | | -0.0016 | -0.0367 | 0.0335 | 0.9283 | -0.018 | -0.0603 | 0.0243 | 0.4033 |
|  | Retired | | -0.004 | -0.0234 | 0.0155 | 0.6888 | -0.0065 | -0.0226 | 0.0097 | 0.4312 |
|  | Homemaker | | 0.0002 | -0.0198 | 0.0202 | 0.981 |  |  |  |  |
|  | Employed (reference) | | 0.000 |  |  |  | 0.000 |  |  |  |
| Living with a partner, no | | | 0.0054 | -0.0014 | 0.0121 | 0.1187 | **0.0129** | 0.0003 | 0.0255 | **0.0455** |
| BMD T-score at baseline | | | 0.0005 | -0.0035 | 0.0044 | 0.8119 | -0.0059 | -0.0124 | 0.0006 | 0.0774 |
| Antiresorptive treatment at baseline, yes | | | 0.0024 | -0.0059 | 0.0107 | 0.5765 | -0.041 | -0.0988 | 0.0168 | 0.1645 |
| Antiresorptive treatment at year 10, yes | | | 0.004 | -0.003 | 0.011 | 0.262 | **0.0298** | 0.0108 | 0.0488 | **0.0021** |
| Falls in past month, yes | | | 0.0043 | -0.0096 | 0.0181 | 0.5454 | 0.0126 | -0.0184 | 0.0437 | 0.4247 |
| Ever confined to bed (immobilized), yes | | | 0.0096 | -0.0032 | 0.0224 | 0.1403 | 0.0107 | -0.0048 | 0.0262 | 0.1771 |
| Ever lost more than 10 pounds, yes | | | 0.0028 | -0.0038 | 0.0095 | 0.4054 | **0.0112** | 0.0002 | 0.0222 | **0.0452** |

CFI= CaMos Frailty Index. BMI= body mass index; MMSE = Mini Mental State Examination; BMD = bone mineral density.

**Supplemental Table S11.**  Effects of incident low-trauma fractures, body mass index and other modifiable and non-modifiable factors on longitudinal changes in the CFI score in CaMos participants **with prior fracture**.

| **Parameter** |  |  | **Estimate** | **95% CI** | | **P-value** |
| --- | --- | --- | --- | --- | --- | --- |
| TIME | 10 year |  | -0.210 | -0.210 | -0.210 | <.0001 |
|  | 5 year |  | -0.152 | -0.182 | -0.122 | <.0001 |
|  | Baseline |  | 0.000 |  |  |  |
| Age, years | >=80 |  | -0.098 | -0.133 | -0.062 | <.0001 |
|  | 65-80 |  | -0.125 | -0.153 | -0.098 | <.0001 |
|  | 50-65 (reference) |  | 0.000 |  |  |  |
| TIME*AGE | 10 year | >=80 | **0.236** | 0.195 | 0.277 | **<.0001** |
|  |  | 65-80 | **0.219** | 0.209 | 0.229 | **<.0001** |
|  |  | 50-65 | 0.000 |  |  |  |
|  | 5 year | >=80 | **0.163** | 0.119 | 0.208 | **<.0001** |
|  |  | 65-80 | **0.179** | 0.148 | 0.210 | **<.0001** |
|  |  | 50-65 | 0.000 |  |  |  |
|  | Baseline (reference) | >=80 | 0.000 |  |  |  |
|  |  | 65-80 | 0.000 |  |  |  |
|  |  | 50-65 | 0.000 |  |  |  |
| Prior Fracture | NHNVF | | **0.014** | 0.003 | 0.025 | **0.012** |
|  | clinical VF | | 0.010 | -0.018 | 0.038 | 0.483 |
|  | Hip | | **0.043** | 0.014 | 0.073 | **0.004** |
|  | None (reference) | | 0.000 |  |  |  |
| Incident Fracture | NHNVF | | 0.002 | -0.028 | 0.032 | 0.884 |
|  | clinical VF | | 0.039 | -0.014 | 0.096 | 0.142 |
|  | Hip | | **0.069** | 0.014 | 0.124 | **0.014** |
|  | None (reference) | | 0.000 |  |  |  |
| Female, yes |  |  | 0.006 | -0.011 | 0.022 | 0.502 |
| Caucasian, yes |  |  | **0.030** | 0.001 | 0.059 | **0.045** |
| BMI, kg/m2 | >=40 | | 0.006 | -0.029 | 0.040 | 0.747 |
|  | 30-40 | | **0.019** | 0.001 | 0.036 | **0.034** |
|  | 25-30 | | 0.005 | -0.008 | 0.018 | 0.416 |
|  | <18.5 | | 0.024 | -0.015 | 0.063 | 0.224 |
|  | 18.5-25 (reference) | | 0.000 |  |  |  |
| Physical activity (kilocal/week, per 1000) | | | **-0.002** | -0.003 | -0.001 | **0.005** |
| Sedentary lifestyle, hours/day | | | 0.000 | -0.002 | 0.002 | 0.836 |
| Total calcium intake (per 1200 mg/day) | | | 0.009 | -0.001 | 0.018 | 0.061 |
| Total vitamin D intake (per 800 IU /day) | | | 0.002 | -0.002 | 0.005 | 0.068 |
| MMSE score (per 3.72 point change) | | | -0.011 | -0.024 | 0.002 | 0.094 |
| SF-36 - Physical subscale (per 5 units change) | | | **-0.031** | -0.034 | -0.028 | **<.0001** |
| SF-36 - Mental subscale (per 5 units change) | | | **-0.015** | -0.018 | -0.011 | **<.0001** |
| Education: University or higher | | | -0.011 | -0.023 | 0.001 | 0.074 |
| Smoking | Never | | -0.012 | -0.033 | 0.008 | 0.218 |
|  | Past | | -0.002 | -0.023 | 0.020 | 0.879 |
|  | Current (reference) | | 0.000 |  |  |  |
| Alcohol, drinks per day | >=3 | | -0.011 | -0.039 | 0.017 | 0.449 |
|  | 1 to 3 | | -0.000 | -0.016 | 0.016 | 0.977 |
|  | >0 to <1 | | -0.004 | -0.015 | 0.007 | 0.509 |
|  | None (reference) | | 0.000 |  |  |  |
| Employment | Unemployed | | **0.033** | 0.004 | 0.063 | **0.027** |
|  | Retired | | -0.006 | -0.029 | 0.016 | 0.584 |
|  | Homemaker | | 0.002 | -0.022 | 0.027 | 0.849 |
|  | Employed (reference) | | 0.000 |  |  |  |
| Living with a partner, no | | | -0.000 | -0.012 | 0.011 | 0.951 |
| BMD T-score at baseline | | | **0.008** | 0.0012 | 0.015 | **0.022** |
| Antiresorptive treatment at baseline , yes | | | -0.006 | -0.021 | 0.009 | 0.445 |
| Antiresorptive treatment at year 10 , yes | | | 0.006 | -0.007 | 0.018 | 0.367 |
| Falls in past month , yes | | | -0.000 | -0.021 | 0.021 | 0.992 |
| Ever confined to bed (immobilized) , yes | | | -0.013 | -0.026 | 0.001 | 0.051 |
| Ever lost more than 10 pounds , yes | | | 0.003 | -0.008 | 0.013 | 0.599 |

CFI= CaMos Frailty Index; NHNVF= non-hip non-clinical vertebral fractures; VF = vertebral fractures ; BMI= body mass index; MMSE = Mini Mental State Examination; BMD = bone mineral density.

**Supplemental Table S12.** Change in the CFI score over time in CaMos participants **with prior fracture**: The effects of low-trauma multiple fractures.

| **Parameter** | | | **Estimate** | **95% CI** | | **P-value** |
| --- | --- | --- | --- | --- | --- | --- |
| Time | 10 year |  | -0.210 | -0.210 | -0.210 | - |
|  | 5 year |  | -0.165 | -0.191 | -0.139 | <.0001 |
|  | Baseline (reference) |  | 0.000 |  |  |  |
| Age, years | >=80 |  | -0.093 | -0.128 | -0.058 | <.0001 |
|  | 65-80 |  | -0.121 | -0.148 | -0.093 | <.0001 |
|  | 50-65 |  | 0.000 |  |  |  |
| Time*Age | 10 year (reference) | >=80 | **0.245** | 0.204 | 0.285 | **<.0001** |
|  |  | 65-80 | **0.219** | 0.210 | 0.229 | **<.0001** |
|  |  | 50-65 | 0 |  |  |  |
|  | 5 year | >=80 | **0.175** | 0.132 | 0.219 | **<.0001** |
|  |  | 65-80 | **0.191** | 0.163 | 0.219 | **<.0001** |
|  |  | 50-65 | 0.000 |  |  |  |
|  | Baseline (reference) | >=80 | 0.000 |  |  |  |
|  |  | 65-80 | 0.000 |  |  |  |
|  |  | 50-65 | 0.000 |  |  |  |
| Prevalent Fracture | Multiple | | **0.033** | 0.012 | 0.055 | **0.002** |
|  | Single | | **0.014** | 0.003 | 0.024 | **0.015** |
|  | None (reference) | | 0.000 |  |  |  |
| Incident Fracture | Multiple | | **0.074** | 0.007 | 0.142 | **0.031** |
|  | Single | | 0.015 | -0.011 | 0.041 | 0.261 |
|  | None (reference) | | 0.000 |  |  |  |
| Caucasian, yes |  |  | 0.027 | -0.005 | 0.058 | 0.097 |
| BMI, kg/m2 | >=40 | | 0.009 | -0.025 | 0.042 | 0.613 |
|  | 30-40 | | **0.020** | 0.003 | 0.038 | **0.025** |
|  | 25-30 | | 0.006 | -0.006 | 0.019 | 0.325 |
|  | <18.5 | | **0.038** | 0.009 | 0.067 | **0.010** |
|  | 18.5-25 (reference) | | 0.000 |  |  |  |
| Physical activity (kilocal/week, per 1000) | | | **-0.001** | -0.002 | 0.000 | **0.006** |
| Sedentary lifestyle, hours/day | | | 0.000 | -0.002 | 0.002 | 0.843 |
| Total calcium intake (per 1200 mg/day) | | | 0.009 | 0.000 | 0.019 | 0.058 |
| Total vitamin D intake (per 800 IU/day) | | | **0.003** | 0.000 | 0.005 | **0.017** |
| MMSE score (per 3.72 point change) | | | -0.011 | -0.024 | 0.001 | 0.079 |
| SF-36 - Physical subscale (per 5 unit change) | | | **-0.031** | -0.034 | -0.029 | **<.0001** |
| SF-36 - Mental subscale (per 5 unit change) | | | **-0.015** | -0.018 | -0.012 | **<.0001** |
| Education: University or higher , yes | | | -0.009 | -0.021 | 0.003 | 0.146 |
| Smoking | Never | | -0.013 | -0.033 | 0.007 | 0.199 |
|  | Past | | -0.003 | -0.023 | 0.018 | 0.808 |
|  | Current (reference) | | 0.000 |  |  |  |
| Alcohol, drinks/ day | >=3 | | 0.003 | -0.044 | 0.049 | 0.914 |
|  | 1 to 3 | | -0.001 | -0.016 | 0.015 | 0.946 |
|  | >0 to <1 | | -0.004 | -0.015 | 0.007 | 0.490 |
|  | None (reference) | | 0.000 |  |  |  |
| Employment | Unemployed | | **0.039** | 0.010 | 0.068 | **0.009** |
|  | Retired | | -0.004 | -0.027 | 0.020 | 0.765 |
|  | Homemaker | | 0.007 | -0.018 | 0.033 | 0.580 |
|  | Employed (reference) | | 0.000 |  |  |  |
| Living with a partner , no | | | 0.001 | -0.010 | 0.012 | 0.856 |
| BMD T-score at baseline | | | **0.007** | 0.000 | 0.014 | **0.039** |
| Antiresorptive treatment at baseline , yes | | | -0.005 | -0.020 | 0.009 | 0.475 |
| Antiresorptive treatment at year 10, yes | | | 0.005 | -0.007 | 0.017 | 0.393 |
| Falls in past month , yes | | | -0.003 | -0.023 | 0.018 | 0.799 |
| Ever confined to bed (immobilized), yes | | | **-0.014** | -0.027 | -0.001 | **0.033** |
| Ever lost more than 10 pounds , yes | | | 0.002 | -0.008 | 0.012 | 0.691 |

CFI= CaMos Frailty Index; BMI= body mass index; MMSE = Mini Mental State Examination;

BMD = bone mineral density.

**Supplemental Table S13.** Descriptive analysis: Loss to follow-up at year 10.

| **Baseline Characteristics** | **Lost To Follow up (N= 3411)** | **Remained in the study (N= 4342)** | **P-value** |
| --- | --- | --- | --- |
| **Demographic Characteristics** |  |  |  |
| Age, mean± SD (range) | 70.51 ± 9.60 (50-103) | 63.65 ± 7.93 (50-91) | <.0001 |
| Females, % (n/N) | 68.43 (2334/3411) | 74.44 (3232/4342) | <.0001 |
| Education (some university or higher), % (n/N) | 19.35 (660/3410) | 28.54 (1239/4342) | <.0001 |
| Caucasians, % (n/N) | 94.46 (3222/3411) | 96.34 (7183/4342) | <.0001 |
| Employment , % (n/N)  Employed (full time, part time)  Homemaker full time  Retired  Other (unemployed, disability, other employment) | 13.08 (446/3409)  19.80 (675/3409)  62.80 (2141/3409)  4.31 (147/3409) | 29.13 (1265/4342)  17.46 (758/4342)  48.57 (2109/4342)  4.84 (210/4342) | <.0001 |
| Living Alone, % (n/N) | 41.45 (1414/3411) | 26.69 (1159/4342) | <.0001 |
| **Anthropometric and Lifestyle Characteristics** |  |  |  |
| Body Mass Index (BMI, kg/m2), mean± SD (range) | 26.86 ± 4.93 (13-51) | 27.22 ± 4.66 (13-64) | 0.0012 |
| BMI, % (n/N)  Underweight: BMI < 18.5  Normal weight: BMI : 18.5 to < 25  Overweight: BMI : 25.0 to < 30  Obesity, class I-III: 30.0 to < 40  Obesity, class IV: >= 40.0 | 2.77 (90/3250)  34.58 (1124/3250)  40.31 (1310/3250)  20.83 (677/3250)  1.51 (49/3250) | 0.94 (40/4274)  32.87 (1405/4274)  42.23 (1805/4274)  22.63 (967/4274)  1.33 (57/4274) | <.0001 |
| Ever lost 10 pounds , % (n/N) | 49.30 (1680/3408) | 51.06 (2217/4342) | 0.123 |
| Exercise - number of kilo-cal/week, mean± SD (range) | 3677 ± 3371 (0-25829) | 4870 ± 3743 (0-61645) | <.0001 |
| Number of sedentary hours/day, mean± SD (range) | 13.93 ± 2.99(5-24) | 13.84 ± 2.92 (5-24) | 0.163 |
| Smoking , % (n/N)  Non-smoker  Past smoker  Current smoker | 42.80 (1460/3411)  40.46 (1380/3411)  16.74 (571/3411) | 49.08 (2130/4340)  38.57 (1674/4340)  12.35 (536/4340) | <.0001 |
| Alcohol consumption - drinks per year, mean± SD (range) | 138.9 ± 312.5 (0-4380) | 159.4 ± 298.5 (0-4380) | 0.0034 |
| Alcohol consumption per day, % (n/N)  None  <= 1  >1 | 49.93 (1703/3411)  20.23 (690/3411)  29.84 (1018/3411) | 36.69 (1593/4342)  23.63 (1026/4342)  35.339.68 (1723/4342) | <.0001 |
| **Baseline Characteristics** | **Lost To Follow up (N= 3411)** | **Remained in the study (N= 4342)** | **P-value** |
| **Bone Health** |  |  |  |
| Calcium intake - mg/day from food and supplements, mean± SD (range) | 994 ± 612 (1.95- 4616) | 1041 ± 621 (0- 6009) | 0.0011 |
| Vitamin D - IU/day from food and supplements, mean± SD (range) | 287.7 ± 854 (0-25436) | 306 ± 1107 (0-37850) | 0.41 |
| BMD T-score at the femoral neck, mean± SD (range) | -1.68 ± 1.04(-5.3 - 4.9) | -1.42 ± 0.98(-4.6 - 3.3) | <.0001 |
| Falls in the past month, % (n/N) | 7.15 (244/3411) | 5.96 (259/4342) | 0.035 |
| Any prior low trauma fractures (excluding head, toe and finger fractures), % (n/N) | 22.0 (747/3398) | 19.1 (827/4338) | 0.0015 |
| Immobilized , % (n/N) | 14.81 (505/3411) | 12.53 (544/4342) | 0.0036 |
| Use of antiresorptive drugs , % (n/N) | 14.42 (492/3411) | 23.84 (1035/4342) | <.0001 |
| **Health-Related Quality of Life** |  |  |  |
| SF-36: Physical subscale score, mean± SD (range) | 43.53 ± 10.99 (7-68) | 48.30 ± 9.35 (10-72; 7706) | <.0001 |
| SF-36: Mental health subscale score, mean± SD (range) | 10.71 ± 1.77 (1-15) | 10.79 ± 1.67 (1-15) | 0.027 |
| **Cognitive Status** |  |  |  |
| MMSE, total score, mean± SD (range) | 27.50 ± 2.71 (1-30) | 28.59 ± 1.85 (1-30) | <.0001 |
| **Frailty Index (FI)** |  |  |  |
| FI score at baseline, mean± SD (range) | 0.178 ± 0.12 (0-0.66) | 0.12 ± 0.09 (0-0.60) | <.0001 |

Exercise includes all types of physical activities; MMSE=Mini Mental State Examination, assessed in participants age 65+.

**Supplemental Table S14.** Differences in the CFI scores between CaMos participants who remained in the study, were lost-to follow-up or died by the end of year 10.

| **CFI scores** | **All participants** | | | **Participants with no prior fractures** | | | **Participants with prior fractures** | | |
| --- | --- | --- | --- | --- | --- | --- | --- | --- | --- |
|  | **Total**  Mean (SD)  [range] | **Women**  Mean (SD)  [range] | **Men**  Mean (SD)  [range] | **Total**  Mean (SD)  [range] | **Women**  Mean (SD)  [range] | **Men**  Mean (SD)  [range] | **Total**  Mean (SD)  [range] | **Women**  Mean (SD)  [range] | **Men**  Mean (SD)  [range] |
| **Baseline** | | | | | | | | | |
| Remained in the study | 0·11 (0·09)  [0·00-0·60] | 0·13 (0·09)  [0·00-0·60] | 0·09 (0·08)  [0·00-0·48] | 0·11 (0·09)  [0·00-0·58] | 0·12 (0·09)  [0·00-0·58] | 0·09 (0·08)  [0·00-0·48] | 0·13 (0·10)  [0·00-0·60] | 0·14 (0·10)  [0·00-0·60] | 0·10 (0·07)  [0·00-0·35] |
| Lost to follow-up | 0·16 (0·11)  [0·00-0·66] | 0·17 (0·12)  [0·00-0·66] | 0·13 (0·10)  [0·00-0·49] | 0·16 (0·11)  [0·00-0·66] | 0·17 (0·12)  [0·00-0·66] | 0·13 (0·10)  [0·00-0·49] | 0·18 (0·12)  [0·00-0·57] | 0·19 (0·12)  [0·01-0·57] | 0·14 (0·10)  [0·00-0·42] |
| Died by the end of follow-up | 0·20 (0·12)  [0·00-0·59] | 0·22 (0·13)  [0·01-0·59] | 0·17 (0·11)  [0·00-0·53] | 0·20 (0·12)  [0·00-0·59] | 0·22 (0·12)  [0·01-0·59] | 0·17 (0·11)  [0·00-0·53] | 0·23 (0·13)  [0·00-0·56] | 0·24 (0·13)  [0·01-0·56] | 0·19 (0·11)  [0·00-0·45] |
| **Year 5** | | | | | | | | | |
| Remained in the study | 0·14 (0·10)  [0·00-0·65] | 0·15 (0·10)  [0·00-0·65] | 0·11 (0·09)  [0·00-0·53] | 0·14 (0·10)  [0·00-0·57] | 0·14 (0·10)  [0·00-0·57] | 0·11 (0·09)  [0·00-0·53] | 0·16 (0·11)  [0·00-0·65] | 0·17 (0·11)  [0·00-0·65] | 0·12 (0·09)  [0·00-0·53] |
| Lost-to follow-up | 0·19 (0·13)  [0·00-0·61] | 0·20 (0·13)  [0·00-0·61] | 0·16 (0·11)  [0·00-0·57] | 0·18 (0·13)  [0·00-0·61] | 0·20 (0·13)  [0·01-0·61] | 0·15 (0·11)  [0·00-0·57] | 0·21 (0·12)  [0·01-0·56] | 0·21 (0·12)  [0·01-0·56] | 0·18 (0·13)  [0·02-0·52] |
| Died by the end of follow-up | 0·24 (0·13)  [0·00-0·62] | 0·27 (0·13)  [0·00-0·62] | 0·20 (0·12)  [0·01-0·54] | 0·24 (0·13)  [0·01-0·61] | 0·26 (0·14)  [0·01-0·61] | 0·19 (0·12)  [0·01-0·50] | 0·27 (0·13)  [0·00-0·62] | 0·28 (0·13)  [0·00-0·62] | 0·23 (0·13)  [0·01-0·54] |
| **Year 10** | | | | | | | | | |
| Remained in the study | 0·12 (0·09)  [0·00-0·53] | 0·12 (0·09)  [0·00-0·53] | 0·10 (0·08)  [0·00-0·45] | 0·12 (0·09)  [0·00-0·52] | 0·12 (0·09)  [0·00-0·52] | 0·10 (0·08)  [0·00-0·45] | 0·14 (0·10)  [0·00-0·53] | 0·15 (0·10)  [0·00-0·53] | 0·10 (0·08)  [0·00-0·40] |
| Lost to follow-up | 0·06 (0·04)  [0·03-0·10] | NA* | 0·07 (0·04)  [0·04-0·10] | 0·06 (0·04)  [0·03-0·10] | NA* | 0·07 (0·04)  [0·04-0·10] | NA | NA | NA |
| Died by the end of follow-up | 0·24 (0·11)  [0·01-0·48] | 0·24 (0·10)  [0·04-0·48] | 0·23 (0·12)  [0·01-0·41] | 0·23 (0·11)  [0·01-0·48] | 0·24 (0·11)  [0·08-0·48] | 0·23 (0·12)  [0·01-0·41] | 0·26 (0·10)  [0·04-0·43] | 0·25 (0·10)  [0·04-0·43] | 0·27 (0·12)  [0·11-0·40] |

SD= standard deviation; NA= not applicable; * Small sample size (N =1).

**Supplemental Table S15.** Missing data analysis: Longitudinal changes in the CFI scores in CaMos participants **with or without prior fracture.**

| **Parameter** | | | **Women** | | | | **Men** | | | |
| --- | --- | --- | --- | --- | --- | --- | --- | --- | --- | --- |
|  |  |  | **Estimate** | **95% CI** | | **P-value** | **Estimate** | **95% CI** | | **P-value** |
| Time | 10 year |  | -0.011 | -0.014 | -0.007 | <.0001 | -0.001 | -0.007 | 0.004 | 0.625 |
|  | 5 year |  | 0.015 | 0.012 | 0.019 | <.0001 | 0.010 | 0.005 | 0.015 | 0.000 |
|  | Baseline |  | 0.000 |  |  |  | 0.000 |  |  |  |
| Age, years | >=80 |  | 0.042 | 0.028 | 0.055 | <.0001 | 0.025 | 0.001 | 0.048 | 0.042 |
|  | 65-80 |  | 0.018 | 0.013 | 0.023 | <.0001 | 0.019 | 0.013 | 0.026 | <.0001 |
|  | 50-65 (reference) |  | 0.000 |  |  |  | 0.000 |  |  |  |
| Time*Age | 10 year | >=80 | **0.030** | 0.006 | 0.053 | **0.013** | **0.076** | 0.039 | 0.114 | **<.0001** |
|  |  | 65-80 | **0.021** | 0.015 | 0.027 | **<.0001** | **0.021** | 0.011 | 0.031 | **<.0001** |
|  |  | 50-65 | 0.000 |  |  |  | 0.000 |  |  |  |
|  | 5 year | >=80 | 0.006 | -0.014 | 0.025 | 0.573 | **0.054** | 0.018 | 0.090 | **0.004** |
|  |  | 65-80 | **0.014** | 0.008 | 0.019 | **<.0001** | **0.022** | 0.013 | 0.030 | **<.0001** |
|  |  | 50-65 | 0.000 |  |  |  | 0.000 |  |  |  |
|  | Baseline (reference) | >=80 | 0.000 |  |  |  | 0.000 |  |  |  |
|  |  | 65-80 | 0.000 |  |  |  | 0.000 |  |  |  |
|  |  | 50-65 | 0.000 |  |  |  | 0.000 |  |  |  |
| Prevalent Fracture | NHNVF | | 0.005 | -0.001 | 0.011 | 0.092 | 0.001 | -0.008 | 0.011 | 0.775 |
|  | clinical VF | | 0.008 | -0.014 | 0.030 | 0.487 | 0.044 | -0.005 | 0.092 | 0.076 |
|  | Hip | | 0.023 | -0.002 | 0.048 | 0.066 | **0.046** | 0.013 | 0.078 | **0.006** |
|  | None (reference) | | 0.000 |  |  |  | 0.000 |  |  |  |
| Incident Fracture | NHNVF | | 0.007 | -0.003 | 0.017 | 0.163 | 0.024 | -0.004 | 0.051 | 0.089 |
|  | clinical VF | | **0.041** | 0.019 | 0.063 | **0.000** | 0.042 | -0.013 | 0.098 | 0.136 |
|  | Hip | | **0.061** | 0.028 | 0.094 | **0.000** | **0.106** | 0.064 | 0.147 | **<.0001** |
|  | None (reference) | | 0.000 |  |  |  | 0.000 |  |  |  |
| Caucasian, yes |  |  | -0.001 | -0.009 | 0.007 | 0.868 | -0.008 | -0.019 | 0.004 | 0.178 |
| BMI, kg/m2 | >=40 | | **0.054** | 0.037 | 0.072 | **<.0001** | **0.082** | 0.037 | 0.127 | **0.000** |
|  | 30-40 | | **0.020** | 0.015 | 0.026 | **<.0001** | **0.020** | 0.011 | 0.028 | **<.0001** |
|  | 25-30 | | **0.007** | 0.003 | 0.011 | **0.000** | **0.011** | 0.005 | 0.018 | **0.001** |
|  | <18.5 | | **-0.013** | -0.025 | -0.001 | **0.040** | **0.045** | -0.014 | 0.104 | **0.137** |
|  | 18.5-25 (reference) | | 0.000 |  |  |  | 0.000 |  |  |  |
| Physical activity (kilocal/week, per 1000) | | | **-0.001** | -0.001 | 0.000 | **0.001** | 0.000 | -0.001 | 0.000 | 0.165 |
| Sedentary lifestyle, hours/day | | | 0.001 | 0.000 | 0.001 | 0.070 | 0.000 | -0.001 | 0.001 | 0.368 |
| Total calcium intake (per 1200 mg/day) | | | 0.003 | 0.000 | 0.007 | 0.060 | 0.001 | -0.005 | 0.006 | 0.834 |
| Total vitamin D intake (per 800IU/day) | | | 0.000 | -0.001 | 0.001 | 0.682 | -0.001 | -0.005 | 0.003 | 0.603 |
| MMSE score (per 3.72 point change) | | | **-0.010** | -0.016 | -0.004 | **0.001** | -0.008 | -0.021 | 0.005 | 0.210 |
| SF-36 - Physical subscale (per 5 unit change) | | | **-0.030** | -0.031 | -0.029 | **<.0001** | **-0.027** | **-0.029** | **-0.025** | **<.0001** |
| SF-36 - Mental subscale (per 5 unit change) | | | **-0.014** | -0.015 | -0.013 | **<.0001** | **-0.014** | **-0.016** | **-0.012** | **<.0001** |
| Education: University or higher, yes | | | **-0.007** | -0.011 | -0.003 | **0.001** | **-0.010** | **-0.016** | **-0.004** | **0.001** |
| Smoking | Never | | **-0.008** | -0.014 | -0.002 | **0.007** | **-0.010** | **-0.023** | **-0.003** | **0.009** |
|  | Past | | -0.004 | -0.010 | 0.002 | 0.224 | -0.010 | -0.015 | 0.003 | 0.213 |
|  | Current (reference) | | 0.000 |  |  |  | 0.000 |  |  |  |
| Alcohol, drinks/day | >=3 | | **-0.008** | **-0.024** | **0.009** | **0.373** | -0.010 | -0.002 | 0.011 | 0.588 |
|  | 1 to 3 | | **-0.007** | -0.013 | -0.002 | **0.012** | -0.010 | -0.006 | 0.010 | 0.681 |
|  | >0 to <1 | | **-0.007** | -0.011 | -0.004 | **0.000** | -0.010 | -0.008 | 0.014 | 0.165 |
|  | None (reference) | | 0.000 |  |  |  | 0.000 |  |  |  |
| Employment | Unemployed | | **0.009** | 0.004 | 0.015 | **0.000** | **0.018** | **0.004** | **0.032** | **0.010** |
|  | Retired | | **0.007** | 0.002 | 0.012 | **0.004** | 0.004 | -0.002 | 0.010 | 0.189 |
|  | Homemaker | | 0.004 | -0.005 | 0.013 | 0.422 | 0.000 | -0.004 | 0.000 | 0.000 |
|  | Employed (reference) | | 0.000 |  |  |  | 0.000 |  |  |  |
| Living with a partner , no | | | 0.004 | -0.001 | 0.008 | 0.093 | 0.002 | -0.006 | 0.010 | 0.548 |
| BMD T-score at baseline | | | 0.001 | -0.001 | 0.003 | 0.489 | -0.002 | -0.006 | 0.003 | 0.459 |
| Antiresorptive treatment at baseline , yes | | | 0.000 | -0.004 | 0.004 | 0.999 | -0.044 | -0.091 | 0.003 | 0.065 |
| Antiresorptive treatment at year 10, yes | | | 0.002 | -0.002 | 0.005 | 0.389 | **0.020** | **0.008** | **0.031** | **0.001** |
| Falls in past month, yes | | | 0.006 | -0.001 | 0.014 | 0.102 | 0.009 | -0.005 | 0.022 | 0.193 |
| Ever confined to bed (immobilized), yes | | | 0.002 | -0.003 | 0.008 | 0.429 | 0.005 | -0.004 | 0.013 | 0.260 |
| Ever lost more than 10 pounds, yes | | | 0.003 | -0.001 | 0.006 | 0.134 | 0.005 | -0.001 | 0.010 | 0.086 |

CFI= CaMos Frailty Index; NHNVF= non-hip non-clinical vertebral fractures; VF = vertebral fractures ; BMI= body mass index; MMSE = Mini Mental State Examination; BMD = bone mineral density.

**Supplemental Table S16.** Missing data analysis: Longitudinal changes in the CFI scores in CaMos participants **without prior fracture.**

| **Parameter** | | | **Women** | | | | **Men** | | | |
| --- | --- | --- | --- | --- | --- | --- | --- | --- | --- | --- |
|  |  |  | **Estimate** | **95% CI** | | **P-value** | **Estimate** | **95% CI** | | **P-value** |
| Time | 10 year |  | -0.011 | -0.015 | -0.007 | <.0001 | -0.001 | -0.008 | 0.005 | 0.673 |
|  | 5 year |  | 0.015 | 0.011 | 0.019 | <.0001 | 0.009 | 0.003 | 0.015 | 0.002 |
|  | Baseline (reference) |  | 0.000 |  |  |  | 0.000 |  |  |  |
| Age, years | >=80 |  | 0.044 | 0.026 | 0.062 | <.0001 | 0.020 | -0.005 | 0.045 | 0.124 |
|  | 65-80 |  | 0.018 | 0.013 | 0.024 | <.0001 | 0.020 | 0.012 | 0.027 | <.0001 |
|  | 50-65 (reference) |  | 0.000 |  |  |  | 0.000 |  |  |  |
| Time*Age | 10 year | >=80 | 0.023 | -0.004 | 0.050 | 0.091 | **0.083** | 0.041 | 0.125 | **<.0001** |
|  |  | 65-80 | **0.022** | 0.016 | 0.029 | **<.0001** | **0.021** | 0.010 | 0.033 | **0.000** |
|  |  | 50-65 | 0.000 |  |  |  | 0.000 |  |  |  |
|  | 5 year | >=80 | 0.011 | -0.014 | 0.035 | 0.395 | **0.069** | 0.031 | 0.106 | **0.000** |
|  |  | 65-80 | **0.014** | 0.009 | 0.020 | **<.0001** | **0.021** | 0.012 | 0.031 | **<.0001** |
|  |  | 50-65 | 0.000 |  |  |  | 0.000 |  |  |  |
|  | Baseline (reference) | >=80 | 0.000 |  |  |  | 0.000 |  |  |  |
|  |  | 65-80 | 0.000 |  |  |  | 0.000 |  |  |  |
|  |  | 50-65 | 0.000 |  |  |  | 0.000 |  |  |  |
| Incident Fracture | NHNVF | | 0.008 | -0.002 | 0.019 | 0.119 | **0.037** | 0.005 | 0.069 | **0.024** |
|  | clinical VF | | **0.047** | 0.020 | 0.073 | **0.001** | 0.031 | -0.028 | 0.090 | 0.306 |
|  | Hip | | **0.064** | 0.020 | 0.109 | **0.005** | **0.095** | 0.045 | 0.146 | **0.000** |
|  | None (reference) | | 0.000 |  |  |  | 0.000 |  |  |  |
| Caucasian, yes |  |  | -0.002 | -0.011 | 0.007 | 0.654 | -0.006 | -0.019 | 0.006 | 0.291 |
| BMI, kg/m2 | >=40 | | **0.062** | 0.041 | 0.083 | **<.0001** | **0.076** | 0.031 | 0.122 | **0.001** |
|  | 30-40 | | **0.020** | 0.015 | 0.026 | **<.0001** | **0.019** | 0.010 | 0.027 | **<.0001** |
|  | 25-30 | | **0.009** | 0.005 | 0.013 | **<.0001** | **0.011** | 0.004 | 0.018 | **0.002** |
|  | <18.5 | | **-0.027** | -0.038 | -0.017 | **<.0001** | 0.045 | -0.020 | 0.110 | 0.173 |
|  | 18.5-25 (reference) | | 0.000 |  |  |  | 0.000 |  |  |  |
| Physical activity (kilocal/week, per 1000) | | | -0.001 | -0.001 | 0.000 | 0.050 | 0.000 | -0.001 | 0.000 | 0.306 |
| Sedentary lifestyle, hours/day | | | **0.001** | 0.000 | 0.002 | **0.032** | 0.000 | -0.001 | 0.001 | 0.491 |
| Total calcium intake (per 1200 mg/day) | | | 0.002 | -0.002 | 0.006 | 0.376 | 0.003 | -0.004 | 0.009 | 0.426 |
| Total vitamin D intake (per 800 /day) | | | 0.000 | -0.001 | 0.001 | 0.759 | -0.004 | -0.008 | 0.001 | 0.140 |
| MMSE score (per 3.72 point change) | | | **-0.009** | -0.016 | -0.002 | **0.016** | -0.010 | -0.025 | 0.004 | 0.169 |
| SF-36 - Physical subscale (per 5 unit change) | | | **-0.029** | -0.031 | -0.028 | **<.0001** | **-0.027** | -0.029 | -0.025 | **<.0001** |
| SF-36 - Mental subscale (per 5 unit change) | | | **-0.014** | -0.016 | -0.013 | **<.0001** | **-0.014** | -0.016 | -0.012 | **<.0001** |
| Education: University or higher , yes | | | **-0.007** | -0.012 | -0.003 | **0.001** | **-0.010** | -0.016 | -0.003 | **0.003** |
| Smoking | Never | | -0.007 | -0.014 | -0.001 | 0.023 | -0.007 | -0.017 | 0.002 | 0.140 |
|  | Past | | -0.003 | -0.010 | 0.003 | 0.308 | -0.001 | -0.010 | 0.008 | 0.848 |
|  | Current (reference) | | 0.000 |  |  |  | 0.000 |  |  |  |
| Alcohol, drinks/ day | >=3 | | -0.017 | -0.035 | 0.001 | 0.068 | 0.000 | -0.011 | 0.012 | 0.978 |
|  | 1 to 3 | | **-0.007** | -0.013 | -0.001 | **0.034** | -0.001 | -0.009 | 0.008 | 0.906 |
|  | >0 to <1 | | **-0.007** | -0.011 | -0.003 | **0.001** | 0.004 | -0.003 | 0.011 | 0.275 |
|  | None (reference) | | 0.000 |  |  |  | 0.000 |  |  |  |
| Employment | Unemployed | | 0.000 | -0.009 | 0.010 | 0.938 | **0.017** | 0.002 | 0.032 | **0.025** |
|  | Retired | | **0.008** | 0.003 | 0.013 | **0.003** | 0.003 | -0.004 | 0.009 | 0.390 |
|  | Homemaker | | **0.009** | 0.003 | 0.014 | **0.002** | **0.000** | -0.004 | 0.000 | **0.000** |
|  | Employed (reference) | | 0.000 |  |  |  | 0.000 |  |  |  |
| Living with a partner, no | | | 0.004 | 0.000 | 0.009 | 0.069 | 0.002 | -0.006 | 0.011 | 0.624 |
| BMD T-score at baseline | | | 0.000 | -0.003 | 0.002 | 0.830 | -0.001 | -0.006 | 0.003 | 0.481 |
| Antiresorptive treatment at baseline , yes | | | 0.001 | -0.003 | 0.006 | 0.495 | **-0.070** | -0.095 | -0.046 | **<.0001** |
| Antiresorptive treatment at year 10, yes | | | 0.001 | -0.003 | 0.005 | 0.763 | **0.021** | 0.008 | 0.035 | **0.002** |
| Falls in past month , yes | | | **0.009** | 0.001 | 0.018 | **0.024** | 0.004 | -0.012 | 0.020 | 0.602 |
| Ever confined to bed (immobilized), yes | | | 0.006 | -0.001 | 0.013 | 0.090 | 0.008 | -0.001 | 0.018 | 0.094 |
| Ever lost more than 10 pounds, yes | | | 0.002 | -0.002 | 0.006 | 0.337 | 0.006 | 0.000 | 0.012 | 0.064 |

CFI= CaMos Frailty Index; NHNVF= non-hip non-clinical vertebral fractures; VF = vertebral fractures ;BMI= body mass index; MMSE = Mini Mental State Examination; BMD = bone mineral density.

**Supplemental Table S17.** Missing data analysis: Longitudinal changes in the CFI scores in CaMos participants **with prior fracture.**

| **Parameter** |  |  | **Estimate** | **95% CI** | | **P-value** |
| --- | --- | --- | --- | --- | --- | --- |
| Time | 10 year |  | -0.005 | -0.014 | 0.003 | 0.192 |
|  | 5 year |  | 0.017 | 0.009 | 0.025 | <.0001 |
|  | Baseline (reference) |  | 0.000 |  |  |  |
| Age, years | >=80 |  | 0.039 | 0.019 | 0.059 | 0.000 |
|  | 65-80 |  | 0.016 | 0.007 | 0.026 | 0.001 |
|  | 50-65(reference) |  | 0.000 |  |  |  |
| Time*Age | 10 year | >=80 | **0.015** | 0.003 | 0.027 | **0.016** |
|  |  | 65-80 | 0.038 | -0.002 | 0.078 | 0.063 |
|  |  | 50-65 | 0.000 |  |  |  |
|  | 5 year | >=80 | **0.012** | 0.001 | 0.023 | **0.037** |
|  |  | 65-80 | 0.001 | -0.030 | 0.032 | 0.962 |
|  |  | 50-65 | 0.000 |  |  |  |
|  | Baseline(reference) | >=80 | 0.000 |  |  |  |
|  |  | 65-80 | 0.000 |  |  |  |
|  |  | 50-65 | 0.000 |  |  |  |
| Prior Fracture | NHNVF | | 0.004 | -0.004 | 0.011 | 0.313 |
|  | clinical VF | | 0.010 | -0.012 | 0.031 | 0.367 |
|  | Hip | | **0.027** | 0.004 | 0.051 | **0.023** |
|  | None(reference) | | 0.000 |  |  |  |
| Incident Fracture | NHNVF | | 0.001 | -0.019 | 0.020 | 0.944 |
|  | clinical VF | | 0.030 | -0.006 | 0.066 | 0.104 |
|  | Hip | | **0.055** | 0.008 | 0.102 | **0.022** |
|  | None(reference) | | 0.000 |  |  |  |
| Female, yes |  |  | 0.010 | 0.000 | 0.020 | 0.051 |
| Caucasian, yes |  |  | -0.003 | -0.019 | 0.014 | 0.752 |
| BMI, kg/m2 | >=40 | | **0.027** | **0.002** | **0.052** | **0.036** |
|  | 30-40 | | **0.023** | **0.011** | **0.035** | **0.000** |
|  | 25-30 | | 0.003 | -0.005 | 0.012 | 0.436 |
|  | <18.5 | | 0.008 | -0.018 | 0.035 | 0.531 |
|  | 18.5-25(reference) | | 0.000 |  |  |  |
| Physical activity (kilocal/week, per 1000) | | | **-0.001** | -0.002 | 0.000 | **0.003** |
| Sedentary lifestyle, hours/day | | | 0.000 | -0.002 | 0.001 | 0.702 |
| Total calcium intake (per 1200 mg/day) | | | 0.006 | -0.002 | 0.013 | 0.136 |
| Total vitamin D intake (per 800IU /day) | | | -0.002 | -0.007 | 0.004 | 0.540 |
| MMSE score (per 3.72 point change) | | | -0.009 | -0.020 | 0.002 | 0.092 |
| SF-36 - Physical subscale (per 5 unit score change) | | | **-0.031** | -0.033 | -0.029 | **<.0001** |
| SF-36 - Mental subscale (per 5 unit score change) | | | **-0.015** | -0.017 | -0.012 | **<.0001** |
| Education: University or higher, yes | | | -0.006 | -0.014 | 0.001 | 0.099 |
| Smoking | Never | | **-0.016** | -0.028 | -0.003 | **0.012** |
|  | Past | | -0.011 | -0.024 | 0.002 | 0.087 |
|  | Current (reference) | | 0.000 | 0.000 | 0.000 | . |
| Alcohol, drinks/day | >=3 | | 0.015 | -0.009 | 0.038 | 0.217 |
|  | 1 to 3 | | -0.004 | -0.014 | 0.007 | 0.519 |
|  | >0 to <1 | | -0.004 | -0.012 | 0.004 | 0.291 |
|  | None (reference) | | 0.000 |  |  |  |
| Employment | Unemployed | | **0.022** | 0.004 | 0.040 | **0.016** |
|  | Retired | | 0.005 | -0.005 | 0.014 | 0.333 |
|  | Homemaker | | **0.014** | 0.001 | 0.026 | **0.035** |
|  | Employed (reference) | | 0.000 |  |  |  |
| Living with a partner, no | | | 0.002 | -0.006 | 0.011 | 0.586 |
| BMD T-score at baseline | | | 0.003 | -0.002 | 0.008 | 0.185 |
| Antiresorptive treatment at baseline , yes | | | -0.003 | -0.012 | 0.006 | 0.541 |
| Antiresorptive treatment at year 10, yes | | | 0.006 | -0.002 | 0.015 | 0.142 |
| Falls in past month, yes | | | 0.001 | -0.012 | 0.014 | 0.915 |
| Ever confined to bed (immobilized), yes | | | -0.008 | -0.016 | 0.000 | 0.064 |
| Ever lost more than 10 pounds, yes | | | 0.003 | -0.004 | 0.011 | 0.351 |

CFI= CaMos Frailty Index; NHNVF= non-hip non-clinical vertebral fractures; VF = vertebral fractures ;BMI= body mass index; MMSE = Mini Mental State Examination; BMD = bone mineral density.

**Supplemental Table S18.** Worst Case Scenario 1 - imputations of the highest hip fracture-related CFI scores for CaMos participants who were lost-to-follow-up: Analyses in the sample **with or without prior fracture.**

| **Parameter** | | | **Women** | | | | **Men** | | | |
| --- | --- | --- | --- | --- | --- | --- | --- | --- | --- | --- |
|  |  |  | **Estimate** | **95% CI** | | **P-value** | **Estimate** | **95% CI** | | **P-value** |
| Time | 10 year |  | -0.0103 | -0.014 | -0.006 | <.0001 | -0.0013 | -0.0071 | 0.0045 | 0.6607 |
|  | 5 year |  | 0.0156 | 0.012 | 0.0192 | <.0001 | 0.0098 | 0.0045 | 0.015 | 0.0003 |
|  | Baseline (reference) |  | 0.000 |  |  |  | 0.000 |  |  |  |
| Age, years | >=80 |  | 0.0449 | 0.0313 | 0.0584 | <.0001 | 0.0223 | -0.0036 | 0.0481 | 0.0912 |
|  | 65-80 |  | 0.0193 | 0.0144 | 0.0241 | <.0001 | 0.0176 | 0.0106 | 0.0245 | <.0001 |
|  | 50-65 (reference) |  | 0.000 |  |  |  | 0.000 |  |  |  |
| Time*Age | 10 year | >=80 | 0.0255 | -0.001 | 0.0521 | 0.0607 | **0.0707** | 0.0303 | 0.111 | **0.0006** |
|  |  | 65-80 | **0.02** | 0.0136 | 0.0265 | **<.0001** | **0.0211** | 0.0107 | 0.0314 | **<.0001** |
|  |  | 50-65 | 0.000 |  |  |  | 0.000 |  |  |  |
|  | 5 year | >=80 | 0.0032 | -0.02 | 0.0264 | 0.787 | **0.0397** | 0.0002 | 0.0792 | **0.049** |
|  |  | 65-80 | **0.0115** | 0.006 | 0.017 | **<.0001** | **0.0219** | 0.0129 | 0.0308 | **<.0001** |
|  |  | 50-65 | 0.000 |  |  |  | 0.000 |  |  |  |
|  | Baseline (reference) | >=80 | 0.000 |  |  |  | 0.000 |  |  |  |
|  |  | 65-80 | 0.000 |  |  |  | 0.000 |  |  |  |
|  |  | 50-65 | 0.000 |  |  |  | 0.000 |  |  |  |
| Prevalent Fracture | NHNVF |  | 0.0038 | -0.002 | 0.0098 | 0.2128 | 0.0032 | -0.0063 | 0.0127 | 0.5142 |
|  | clinical VF |  | 0.0034 | -0.018 | 0.0255 | 0.7649 | **0.0698** | 0.0439 | 0.0957 | **<.0001** |
|  | Hip |  | 0.0244 | -0.004 | 0.0531 | 0.0951 | **0.0477** | 0.0143 | 0.0812 | **0.0052** |
|  | None (reference) |  | 0.000 |  |  |  | 0.000 |  |  |  |
| Incident Fracture | NHNVF |  | **0.012** | 0.0018 | 0.0223 | **0.0216** | **0.0307** | 0.001 | 0.0605 | **0.043** |
|  | clinical VF |  | **0.0473** | 0.0235 | 0.071 | **<.0001** | 0.043 | -0.0128 | 0.0987 | 0.1312 |
|  | Hip |  | **0.0682** | 0.0328 | 0.1035 | **0.0002** | **0.1054** | 0.0635 | 0.1473 | **<.0001** |
|  | None (reference) |  | 0.000 |  |  |  | 0.000 |  |  |  |
| Caucasian, yes |  |  | -0.001 | -0.009 | 0.0074 | 0.8111 | -0.0063 | -0.0179 | 0.0053 | 0.2856 |
| BMI, kg/m2 | >=40 |  | **0.0566** | 0.0381 | 0.0752 | **<.0001** | **0.0799** | 0.031 | 0.1287 | **0.0013** |
|  | 30-40 |  | **0.021** | 0.0156 | 0.0263 | **<.0001** | **0.0207** | 0.0121 | 0.0294 | **<.0001** |
|  | 25-30 |  | **0.0078** | 0.0037 | 0.0118 | **0.0002** | **0.0121** | 0.0056 | 0.0185 | **0.0003** |
|  | <18.5 |  | -0.0109 | -0.023 | 0.0018 | 0.0915 | 0.0514 | -0.0341 | 0.1368 | 0.2387 |
|  | 18.5-25 (reference) |  | 0.000 |  |  |  | 0.000 |  |  |  |
| Physical Activity(kilocal/week, per 1000) | | | **-0.0009** | -0.001 | -0.000 | **0.0019** | -0.0004 | -0.0009 | 0.0001 | 0.1455 |
| Sedentary lifestyle, hours/day | | | 0.0004 | -0.000 | 0.0011 | 0.205 | -0.0005 | -0.0015 | 0.0004 | 0.2769 |
| Total calcium intake (per 1200 mg/day) | | | 0.0037 | -0.000 | 0.0076 | 0.0558 | -0.0005 | -0.0063 | 0.0052 | 0.8553 |
| Total vitamin D intake (per 800 /day) | | | -0.0003 | -0.001 | 0.0008 | 0.6352 | -0.0014 | -0.0055 | 0.0027 | 0.5039 |
| MMSE score (per 3.72 point change) | | | **-0.01** | -0.017 | -0.003 | **0.0045** | -0.0047 | -0.0175 | 0.0082 | 0.4754 |
| SF-36 - Physical subscale score (per 5 unit change) | | | **-0.0296** | -0.030 | -0.028 | **<.0001** | **-0.0269** | -0.0288 | -0.025 | **<.0001** |
| SF-36 - Mental subscale score (per 5 unit change) | | | **-0.0145** | -0.016 | -0.013 | **<.0001** | **-0.0141** | -0.0162 | -0.0121 | **<.0001** |
| Education: University or higher, yes | | | **-0.0062** | -0.010 | -0.002 | **0.0026** | **-0.0111** | -0.0169 | -0.0053 | **0.0002** |
| Smoking | Never |  | **-0.009** | -0.015 | -0.003 | **0.0033** | **-0.0124** | -0.0225 | -0.0024 | **0.0149** |
|  | Past |  | -0.0047 | -0.011 | 0.0015 | 0.1398 | -0.006 | -0.0156 | 0.0036 | 0.2212 |
|  | Current (reference) |  | 0.000 |  |  |  | 0.000 |  |  |  |
| Alcohol, drinks/day | >=3 |  | -0.012 | -0.029 | 0.0047 | 0.1591 | -0.0002 | -0.0109 | 0.0106 | 0.978 |
|  | 1 to 3 |  | **-0.0069** | -0.013 | -0.001 | **0.0186** | -0.0004 | -0.0087 | 0.0079 | 0.9247 |
|  | >0 to <1 |  | **-0.0073** | -0.011 | -0.003 | **0.0004** | 0.004 | -0.0029 | 0.0109 | 0.259 |
|  | None (reference) |  | 0 |  |  |  | 0.000 |  |  |  |
| Employment | Unemployed |  | 0.0034 | -0.006 | 0.0131 | 0.4907 | **0.0212** | 0.0067 | 0.0356 | **0.0041** |
|  | Retired |  | **0.0073** | 0.0023 | 0.0122 | **0.0041** | 0.0052 | -0.0011 | 0.0115 | 0.1062 |
|  | Homemaker |  | **0.0087** | 0.0033 | 0.0141 | **0.0017** | 0.000 |  |  |  |
|  | Employed (reference) |  | 0.000 |  |  |  | 0.000 |  |  |  |
| Living with a partner, no | | | 0.0027 | -0.002 | 0.007 | 0.2197 | 0.0029 | -0.005 | 0.0107 | 0.4726 |
| BMD T-score at baseline | | | 0.0008 | -0.001 | 0.0029 | 0.4403 | 0.0002 | -0.0038 | 0.0042 | 0.9246 |
| Antiresorptive treatment at baseline , yes | | | 0.0009 | -0.003 | 0.0049 | 0.6684 | **-0.0447** | -0.0891 | -0.0004 | **0.0481** |
| Antiresorptive treatment at year 10, yes | | | 0.0015 | -0.002 | 0.0054 | 0.4453 | **0.0211** | 0.0093 | 0.033 | **0.0005** |
| Falls in past month, yes | | | 0.0065 | -0.001 | 0.0142 | 0.0966 | 0.0068 | -0.0067 | 0.0204 | 0.3228 |
| Ever confined to bed (immobilized), yes | | | 0.0034 | -0.002 | 0.0092 | 0.2529 | 0.0053 | -0.0031 | 0.0138 | 0.2126 |
| Ever lost more than 10 pounds, yes | | | 0.0029 | -0.001 | 0.0066 | 0.1142 | 0.0044 | -0.0013 | 0.0101 | 0.1285 |

CFI= CaMos Frailty Index; NHNVF= non-hip non-vertebral fractures; VF = vertebral fractures;BMI= body mass index; MMSE = Mini Mental State Examination; BMD = bone mineral density.

**Supplemental Table S19.** Worst Case Scenario 1 - imputations of the highest hip fracture-related CFI scores for CaMos participants who were lost-to-follow-up: Analyses in the sample **without prior fracture.**

| **Parameter** | | | **Women** | | | | **Men** | | | |
| --- | --- | --- | --- | --- | --- | --- | --- | --- | --- | --- |
|  |  |  | **Estimate** | **95% CI** | | **P-value** | **Estimate** | **95% CI** | | **P-value** |
| Time | 10 year |  | -0.0061 | -0.017 | 0.0043 | 0.2508 | 0.0008 | -0.0122 | 0.0138 | 0.9017 |
|  | 5 year |  | 0.0206 | 0.0103 | 0.0309 | <.0001 | 0.0122 | 0.001 | 0.0235 | 0.0329 |
|  | Baseline(reference) |  | 0.000 |  |  |  | 0.000 | 0.00 |  |  |
| Age, years | >=80 |  | 0.041 | 0.018 | 0.064 | 0.0005 | 0.0503 | -0.0123 | 0.113 | 0.1152 |
|  | 65-80 |  | 0.0193 | 0.0075 | 0.031 | 0.0013 | 0.0103 | -0.0062 | 0.0267 | 0.2205 |
|  | 50-65(reference) |  | 0.000 |  |  |  | 0.000 |  |  |  |
| Time*Age | 10 year | >=80 | 0.0306 | -0.016 | 0.0774 | 0.2001 | 0.047 | -0.0273 | 0.1212 | 0.2154 |
|  |  | 65-80 | 0.0124 | -0.003 | 0.0276 | 0.11 | 0.0186 | -0.0035 | 0.0407 | 0.0995 |
|  |  | 50-65 | 0.000 |  |  |  | 0.000 |  |  |  |
|  | 5 year | >=80 | -0.0084 | -0.045 | 0.0286 | 0.6574 | -0.0095 | -0.0804 | 0.0615 | 0.7937 |
|  |  | 65-80 | 0.0026 | -0.011 | 0.0166 | 0.7192 | **0.028** | 0.007 | 0.049 | **0.009** |
|  |  | 50-65 | 0.000 |  |  |  | 0.000 |  |  |  |
|  | Baseline(reference) | >=80 | 0.000 |  |  |  | 0.000 |  |  |  |
|  |  | 65-80 | 0.000 |  |  |  | 0.000 |  |  |  |
|  |  | 50-65 | 0.000 |  |  |  | 0.000 |  |  |  |
| Incident Fracture | NHNVF | | 0.005 | -0.018 | 0.0282 | 0.6693 | 0.0013 | -0.0531 | 0.0558 | 0.9617 |
|  | clinical VF | | 0.0292 | -0.012 | 0.0704 | 0.166 | 0.0763 | -0.0333 | 0.1859 | 0.1724 |
|  | Hip | | **0.0665** | 0.0126 | 0.1204 | **0.0156** | **0.111** | 0.0609 | 0.161 | **<.0001** |
|  | None(reference) | | 0.000 |  |  |  | 0.000 |  |  |  |
| Caucasian, yes |  |  | -0.0068 | -0.029 | 0.0164 | 0.5664 | 0.0005 | -0.0279 | 0.0289 | 0.9734 |
| BMI, kg/m2 | >=40 |  | 0.0159 | -0.010 | 0.0423 | 0.2382 | **0.0341** | 0.0069 | 0.0614 | **0.0141** |
|  | 30-40 |  | **0.0214** | 0.0078 | 0.035 | **0.002** | 0.0165 | -0.0027 | 0.0356 | 0.0923 |
|  | 25-30 |  | 0.001 | -0.008 | 0.0103 | 0.839 | 0.000 |  |  |  |
|  | <18.5 |  | 0.0119 | -0.016 | 0.0397 | 0.399 | 0.000 |  |  |  |
|  | 18.5-25(reference) |  | 0.000 |  |  |  | 0.000 |  |  |  |
| Physical Activity(kilocal/week, per 1000) | | | **-0.0014** | -0.003 | -1E-04 | **0.0382** | -0.0005 | -0.0014 | 0.0004 | 0.2477 |
| Sedentary lifestyle, hours/day | | | -0.0002 | -0.002 | 0.0015 | 0.8318 | -0.0015 | -0.0037 | 0.0006 | 0.1674 |
| Total calcium intake (per 1200 mg/day) | | | **0.0108** | 0.0022 | 0.0194 | **0.0136** | -0.0112 | -0.0256 | 0.0032 | 0.1259 |
| Total vitamin D intake (per 800 IU/day) | | | -0.0021 | -0.008 | 0.0043 | 0.5187 | 0.0067 | -0.017 | 0.0305 | 0.5772 |
| MMSE score (per 3.72 point change) | | | -0.0124 | -0.026 | 0.001 | 0.07 | 0.0041 | -0.013 | 0.0211 | 0.6392 |
| SF-36 - Physical subscale score (per 5 units change) | | | **-0.0322** | -0.035 | -0.03 | **<.0001** | **-0.0274** | -0.0326 | -0.022 | **<.0001** |
| SF-36 - Mental subscale score (per 5 units change) | | | **-0.0144** | -0.017 | -0.012 | **<.0001** | **-0.0169** | -0.0221 | -0.012 | **<.0001** |
| Education: University or higher, yes | | | -0.0081 | -0.017 | 0.001 | 0.0823 | -0.007 | -0.0214 | 0.0074 | 0.3437 |
| Smoking | Never |  | -0.011 | -0.024 | 0.0025 | 0.1106 | **-0.0358** | -0.0681 | -0.004 | **0.0294** |
|  | Past |  | -0.0068 | -0.021 | 0.0073 | 0.3435 | -0.0274 | -0.0602 | 0.0054 | 0.1014 |
|  | Current(reference) |  | 0.000 |  |  |  | 0.000 |  |  |  |
| Alcohol, drinks per day | >=3 |  | 0.0333 | -0.000 | 0.0667 | 0.0504 | 0.0029 | -0.0258 | 0.0316 | 0.8425 |
|  | 1 to 3 |  | -0.0048 | -0.018 | 0.0084 | 0.475 | 0.0083 | -0.0145 | 0.0312 | 0.4752 |
|  | >0 to <1 |  | -0.0051 | -0.014 | 0.0042 | 0.2804 | 0.0015 | -0.0197 | 0.0226 | 0.8921 |
|  | None(reference) |  | 0 |  |  |  | 0.000 |  |  |  |
| Employment | Unemployed |  | 0.0231 | 0.0003 | 0.0459 | 0.0472 | 0.0316 | -0.0009 | 0.064 | 0.0565 |
|  | Retired |  | 0.0001 | -0.012 | 0.0119 | 0.9845 | 0.0123 | -0.0048 | 0.0294 | 0.1584 |
|  | Homemaker |  | 0.0069 | -0.007 | 0.0208 | 0.3309 | 0.000 |  |  |  |
|  | Employed (reference) |  | 0.000 |  |  |  | 0.000 |  |  |  |
| Living with a partner, no | | | 0.0014 | -0.008 | 0.0111 | 0.7801 | 0.0183 | -0.0038 | 0.0403 | 0.1043 |
| BMD T-score at baseline | | | 0.0031 | -0.002 | 0.008 | 0.2257 | 0.0021 | -0.0089 | 0.0131 | 0.7045 |
| Antiresorptive treatment at baseline , yes | | | -0.002 | -0.012 | 0.0077 | 0.6916 | -0.0733 | -0.3681 | 0.2214 | 0.6258 |
| Antiresorptive treatment at year 10, yes | | | 0.004 | -0.006 | 0.0135 | 0.416 | 0.0191 | -0.0035 | 0.0417 | 0.0982 |
| Falls in past month, yes | | | -0.005 | -0.021 | 0.0108 | 0.5363 | 0.0225 | -0.0041 | 0.049 | 0.0972 |
| Ever confined to bed (immobilized), yes | | | **-0.0107** | -0.021 | -7E-04 | **0.0364** | -0.0052 | -0.0217 | 0.0114 | 0.5407 |
| Ever lost more than 10 pounds, yes | | | 0.0049 | -0.003 | 0.0133 | 0.2536 | -0.0051 | -0.0193 | 0.0092 | 0.4853 |

CFI= CaMos Frailty Index; NHNVF= non-hip non-vertebral fractures; VF = vertebral fractures;BMI= body mass index; MMSE = Mini Mental State Examination; BMD = bone mineral density.

**Supplemental Table S20.** Worst Case Scenario 1 - imputations of the highest hip fracture-related CFI scores for CaMos participants who were lost-to-follow-up: Analyses in the sample **with prior fracture.**

| **Parameter** |  |  | **Estimate** | **95% CI** | | **P-value** |
| --- | --- | --- | --- | --- | --- | --- |
| Time | 10 year |  | -0.0042 | -0.0126 | 0.0041 | 0.3219 |
|  | 5 year |  | 0.0185 | 0.0104 | 0.0267 | <.0001 |
|  | Baseline(reference) |  | 0.000 | 0.00 | 0.00 | . |
| Age, years | >=80 |  | 0.0436 | 0.0233 | 0.0638 | <.0001 |
|  | 65-80 |  | 0.0184 | 0.0085 | 0.0283 | 0.0003 |
|  | 50-65(reference) |  | 0.000 | 0.00 | 0.00 | . |
| Time*Age | 10 year | >=80 | 0.0303 | -0.0119 | 0.0725 | 0.1588 |
|  |  | 65-80 | **0.0133** | 0.0006 | 0.026 | **0.0409** |
|  |  | 50-65 | 0.000 |  |  |  |
|  | 5 year | >=80 | -0.0075 | -0.0408 | 0.0259 | 0.6608 |
|  |  | 65-80 | 0.0078 | -0.0038 | 0.0195 | 0.1884 |
|  |  | 50-65 | 0.000 |  |  |  |
|  | Baseline(reference) | >=80 | 0.000 |  |  |  |
|  |  | 65-80 | 0.000 |  |  |  |
|  |  | 50-65 | 0.000 |  |  |  |
| Incident Fracture | NHNVF | | 0.0036 | -0.0178 | 0.0251 | 0.7406 |
|  | clinical VF | | 0.0324 | -0.007 | 0.0719 | 0.1068 |
|  | Hip | | **0.0721** | 0.0236 | 0.1206 | **0.0036** |
|  | None(reference) | | 0.000 |  |  |  |
| Prevalent Fracture | NHNVF | | 0.0045 | -0.0032 | 0.0121 | 0.2523 |
|  | clinical VF | | 0.0093 | -0.0141 | 0.0327 | 0.4354 |
|  | Hip | | **0.0273** | 0.0008 | 0.0537 | **0.0436** |
|  | None(reference) | | 0.000 |  |  |  |
| Female, yes |  |  | 0.0072 | -0.003 | 0.0174 | 0.1672 |
| Caucasian, yes |  |  | -0.0019 | -0.019 | 0.0152 | 0.8276 |
| BMI, kg/m2 | >=40 |  | 0.0209 | -0.0053 | 0.0471 | 0.1182 |
|  | 30-40 |  | **0.0236** | 0.0113 | 0.036 | **0.0002** |
|  | 25-30 |  | 0.0047 | -0.004 | 0.0133 | 0.2936 |
|  | <18.5 |  | 0.0121 | -0.0141 | 0.0384 | 0.3657 |
|  | 18.5-25(reference) |  | 0.000 |  |  |  |
| Physical Activity(kilocal/week, per 1000) | | | **-0.001** | -0.0018 | -0.0003 | **0.0076** |
| Sedentary lifestyle, hours/day | | | -0.0005 | -0.0019 | 0.0009 | 0.5111 |
| Total calcium intake (per 1200 mg/day) | | | 0.0067 | -0.0009 | 0.0144 | 0.0836 |
| Total vitamin D intake (per 800IU /day) | | | -0.0021 | -0.0075 | 0.0032 | 0.4336 |
| MMSE score (per 3.72 point change) | | | -0.0098 | -0.0217 | 0.0021 | 0.1063 |
| SF-36 - Physical subscale sclore (per 5 units change) | | | **-0.0313** | -0.0337 | -0.0288 | **<.0001** |
| SF-36 - Mental subscale sclore (per 5 units change) | | | **-0.0148** | -0.0173 | -0.0124 | **<.0001** |
| Education: University or higher, yes | | | **-0.0085** | -0.0161 | -0.0009 | **0.0283** |
| Smoking | Never |  | **-0.0151** | -0.0282 | -0.0021 | **0.0233** |
|  | Past |  | -0.0106 | -0.024 | 0.0027 | 0.1193 |
|  | Current (reference) |  | 0.000 |  |  |  |
| Alcohol, drinks/ day >=3 | |  | 0.0115 | -0.0089 | 0.032 | 0.2689 |
|  | 1 to 3 |  | -0.0024 | -0.0136 | 0.0089 | 0.683 |
|  | >0 to <1 |  | -0.0038 | -0.0121 | 0.0045 | 0.3716 |
|  | None(reference) |  | 0 |  |  |  |
| Employment | Unemployed |  | **0.0265** | 0.0076 | 0.0454 | **0.0059** |
|  | Retired |  | 0.0038 | -0.006 | 0.0136 | 0.4438 |
|  | Homemaker |  | 0.0089 | -0.004 | 0.0217 | 0.1771 |
|  | Employed (reference) |  | 0.000 |  |  |  |
| Living with a partner, no | | | 0.0025 | -0.0063 | 0.0113 | 0.5812 |
| BMD T-score at baseline | | | 0.0036 | -0.001 | 0.0082 | 0.1216 |
| Antiresorptive treatment at baseline , yes | | | -0.0009 | -0.0105 | 0.0087 | 0.86 |
| Antiresorptive treatment at year 10, yes | | | 0.0066 | -0.0022 | 0.0155 | 0.1428 |
| Falls in past month, yes | | | 0.002 | -0.0121 | 0.016 | 0.7824 |
| Ever confined to bed (immobilized), yes | | | -0.0083 | -0.0169 | 0.0002 | 0.0561 |
| Ever lost more than 10 pounds, yes | | | 0.0028 | -0.0045 | 0.0102 | 0.4527 |

**Supplemental Table S21.** Worst Case Scenario 2 - imputations of the upper theoretical CFI limit for CaMos participants who were lost-to-follow-up: Analyses in the sample **with or without prior fracture**.

| P**arameter** | | | **Women** | | | | **Men** | | | |
| --- | --- | --- | --- | --- | --- | --- | --- | --- | --- | --- |
|  |  |  | **Estimate** | **95% CI** | | **P-value** | **Estimate** | **95% CI** | | **P-value** |
| Time | 10 year |  | -0.0103 | -0.0144 | -0.006 | <.0001 | -0.0013 | -0.0071 | 0.0045 | 0.6621 |
|  | 5 year |  | 0.0156 | 0.012 | 0.0192 | <.0001 | 0.0098 | 0.0045 | 0.015 | 0.0003 |
|  | Baseline(reference) |  | 0 |  |  |  | 0 |  |  |  |
| Age, years | >=80 |  | 0.0448 | 0.0312 | 0.0583 | <.0001 | 0.022 | -0.0038 | 0.0478 | 0.0942 |
|  | 65-80 |  | 0.0191 | 0.0142 | 0.024 | <.0001 | 0.0176 | 0.0106 | 0.0246 | <.0001 |
|  | 50-65(reference) |  | 0 |  |  |  | 0 |  |  |  |
| Time*Age | 10 year | >=80 | 0.0255 | -0.0011 | 0.0521 | **0.0606** | 0.0707 | 0.0303 | 0.111 | **0.0006** |
|  |  | 65-80 | 0.0204 | 0.0139 | 0.0269 | **<.0001** | 0.0221 | 0.0114 | 0.0328 | **<.0001** |
|  |  | 50-65 | 0 |  |  |  | 0 |  |  |  |
|  | 5 year | >=80 | 0.0032 | -0.02 | 0.0264 | 0.7868 | 0.0397 | 0.0002 | 0.0792 | **0.049** |
|  |  | 65-80 | 0.0115 | 0.006 | 0.017 | **<.0001** | 0.0219 | 0.0129 | 0.0308 | **<.0001** |
|  |  | 50-65 | 0 |  |  |  | 0 |  |  |  |
|  | Baseline(reference) | >=80 | 0 |  |  |  | 0 |  |  |  |
|  |  | 65-80 | 0 |  |  |  | 0 |  |  |  |
|  |  | 50-65 | 0 |  |  |  | 0 |  |  |  |
| Prevalent Fracture | Non Hip-nonVF | | 0.0038 | -0.0022 | 0.0097 | 0.2181 | 0.0029 | -0.0067 | 0.0124 | 0.5527 |
|  | clinical VF | | 0.0033 | -0.0188 | 0.0254 | 0.771 | 0.0679 | 0.0409 | 0.0949 | <.0001 |
|  | Hip | | 0.0244 | -0.0042 | 0.0531 | 0.0944 | 0.0477 | 0.0151 | 0.0804 | 0.0042 |
|  | None(reference) | | 0 |  |  |  | 0 |  |  |  |
| Incident Fracture | Non Hip-nonVF | | 0.012 | 0.0017 | 0.0223 | **0.0218** | 0.0306 | 0.0008 | 0.0604 | **0.0441** |
|  | clinical VF | | 0.0473 | 0.0236 | 0.0711 | **<.0001** | 0.042 | -0.0135 | 0.0976 | 0.1381 |
|  | Hip | | 0.0681 | 0.0328 | 0.1034 | **0.0002** | 0.1055 | 0.0637 | 0.1473 | **<.0001** |
|  | None(reference) | | 0 |  |  |  | 0 |  |  |  |
| Caucasian, yes |  |  | -0.001 | -0.0094 | 0.0075 | 0.8182 | -0.0058 | -0.0175 | 0.0058 | 0.3288 |
| BMI, kg/m2 | >=40 |  | 0.0569 | 0.0383 | 0.0754 | **<.0001** | 0.0807 | 0.0309 | 0.1304 | **0.0015** |
|  | 30-40 |  | 0.0213 | 0.0159 | 0.0267 | **<.0001** | 0.0212 | 0.0125 | 0.0299 | **<.0001** |
|  | 25-30 |  | 0.0078 | 0.0037 | 0.0118 | **0.0002** | 0.0126 | 0.006 | 0.0192 | **0.0002** |
|  | <18.5 |  | -0.0108 | -0.0234 | 0.0018 | 0.0943 | 0.0498 | -0.0336 | 0.1331 | 0.2418 |
|  | 18.5-25(reference) |  | 0 |  |  |  | 0 |  |  |  |
| Physical Activity(kilocal/week, per 1000) | | | -0.0009 | -0.0015 | -4E-04 | **0.0015** | -0.0004 | -0.001 | 0.0001 | 0.1204 |
| Sedentary lifestyle, hours/day | | | 0.0004 | -0.0003 | 0.0011 | 0.2154 | -0.0005 | -0.0014 | 0.0005 | 0.303 |
| Total calcium intake (per 1200 mg/day) | | | 0.0037 | -0.0001 | 0.0075 | 0.0573 | -0.0008 | -0.0066 | 0.005 | 0.7861 |
| Total vitamin D intake (per 800 /day) | | | -0.0002 | -0.0013 | 0.0008 | 0.6561 | -0.0014 | -0.0056 | 0.0027 | 0.491 |
| MMSE score (per 3.72 point change) | | | -0.0098 | -0.0167 | -0.003 | **0.0058** | -0.0041 | -0.0171 | 0.0088 | 0.531 |
| SF-36 - Physical subscale sclore (per 5 units change) | | | -0.0296 | -0.0307 | -0.028 | **<.0001** | -0.0268 | -0.0287 | -0.025 | **<.0001** |
| SF-36 - Mental subscale sclore (per 5 units change) | | | -0.0145 | -0.0156 | -0.013 | **<.0001** | -0.0141 | -0.0162 | -0.012 | **<.0001** |
| Education: University or higher, yes | | | -0.0063 | -0.0103 | -0.002 | **0.0022** | -0.0114 | -0.0172 | -0.006 | **0.0001** |
| Smoking | Never |  | -0.0089 | -0.0149 | -0.003 | **0.0037** | -0.0139 | -0.0243 | -0.003 | **0.0096** |
|  | Past |  | -0.0047 | -0.0109 | 0.0015 | 0.1379 | -0.0075 | -0.0176 | 0.0027 | 0.1498 |
|  | Current(reference) |  | 0 |  |  |  | 0 |  |  |  |
| Alcohol, drinks/ day | >=3 |  | -0.0119 | -0.0286 | 0.0048 | 0.1629 | -0.001 | -0.012 | 0.0099 | 0.8531 |
|  | 1 to 3 |  | -0.0069 | -0.0126 | -0.001 | **0.0198** | -0.0012 | -0.0098 | 0.0073 | 0.7738 |
|  | >0 to <1 |  | -0.0071 | -0.0112 | -0.003 | **0.0005** | 0.0033 | -0.0038 | 0.0104 | 0.366 |
|  | None(reference) |  | 0 |  |  |  | 0 |  |  |  |
| Employment | Unemployed |  | 0.0034 | -0.0063 | 0.0131 | 0.4905 | 0.0215 | 0.0071 | 0.036 | **0.0035** |
|  | Retired |  | 0.0073 | 0.0024 | 0.0123 | **0.0038** | 0.0054 | -0.0009 | 0.0118 | 0.0917 |
|  | Homemaker |  | 0.0086 | 0.0032 | 0.014 | **0.0017** |  |  |  |  |
|  | Employed(reference) |  | 0 |  |  |  | 0 |  |  |  |
| Living alone, yes |  |  | 0.0029 | -0.0015 | 0.0072 | 0.1937 | 0.0027 | -0.0051 | 0.0106 | 0.4966 |
| BMD T-score at baseline | | | 0.0008 | -0.0012 | 0.0029 | 0.4313 | 0.0003 | -0.0037 | 0.0043 | 0.8784 |
| Antiresorptive treatment at baseline , yes | | | 0.0008 | -0.0031 | 0.0048 | 0.6806 | -0.0443 | -0.0895 | 0.0009 | 0.0546 |
| Antiresorptive treatment at year 10 , yes | | | 0.0015 | -0.0024 | 0.0054 | 0.4655 | 0.0231 | 0.0105 | 0.0357 | **0.0003** |
| Falls in past month , yes | | | 0.0065 | -0.0012 | 0.0142 | 0.0965 | 0.0069 | -0.0067 | 0.0205 | 0.3196 |
| Ever confined to bed (immobilized), yes | | | 0.0033 | -0.0025 | 0.0091 | 0.2613 | 0.0053 | -0.0031 | 0.0137 | 0.2167 |
| Ever lost more than 10 pounds, yes | | | 0.0028 | -0.0008 | 0.0065 | 0.1318 | 0.0041 | -0.0016 | 0.0099 | 0.1554 |

CFI= CaMos Frailty Index; NHNVF= non-hip non-vertebral fractures; VF = vertebral fractures;BMI= body mass index; MMSE = Mini Mental State Examination; BMD = bone mineral density.

**Supplemental Table S22.** Worst Case Scenario 2 - imputations of the upper theoretical CFI limit for CaMos participants who were lost-to-follow-up: Analyses in the sample **without prior fracture**.

| Parameter | | | **Women** | | | | **Men** | | | |
| --- | --- | --- | --- | --- | --- | --- | --- | --- | --- | --- |
|  |  |  | **Estimate** | **95% CI** | | **P-value** | **Estimate** | **95% CI** | | **P-value** |
| TIME | 10 year | | -0.0103 | -0.0144 | -0.0062 | <.0001 | -0.0013 | -0.0071 | 0.0045 | 0.658 |
|  | 5 year | | 0.0156 | 0.012 | 0.0192 | <.0001 | 0.0098 | 0.0045 | 0.015 | 0.0003 |
|  | Baseline(reference) | | 0 |  |  |  | 0 |  |  |  |
| Age, years | >=80 | | 0.046 | 0.0323 | 0.0597 | <.0001 | 0.0252 | -0.0034 | 0.0538 | 0.0842 |
|  | 65-80 | | 0.0194 | 0.0145 | 0.0243 | <.0001 | 0.0176 | 0.0106 | 0.0246 | <.0001 |
|  | 50-65(reference) | | 0 |  |  |  | 0 |  |  |  |
| TIME*AGE | 10 year | >=80 | 0.0254 | -0.0012 | 0.052 | **0.0615** | 0.0707 | 0.0303 | 0.111 | **0.0006** |
|  |  | 65-80 | 0.0201 | 0.0137 | 0.0265 | **<.0001** | 0.0206 | 0.0104 | 0.0309 | **<.0001** |
|  |  | 50-65 | 0 |  |  |  | 0 |  |  |  |
|  | 5 year | >=80 | 0.0032 | -0.02 | 0.0264 | 0.7888 | 0.0397 | 0.0002 | 0.0792 | **0.0489** |
|  |  | 65-80 | 0.0115 | 0.006 | 0.017 | **<.0001** | 0.0218 | 0.0129 | 0.0308 | **<.0001** |
|  |  | 50-65 | 0 |  |  |  | 0 |  |  |  |
|  | Baseline(reference) | >=80 | 0 |  |  |  | 0 |  |  |  |
|  |  | 65-80 | 0 |  |  |  | 0 |  |  |  |
|  |  | 50-65 | 0 |  |  |  | 0 |  |  |  |
| Incident Fracture | NHNVF | | 0.0121 | 0.0019 | 0.0224 | **0.0204** | 0.0313 | 0.0016 | 0.061 | **0.0387** |
|  | clinical VF | | 0.0475 | 0.0238 | 0.0711 | **<.0001** | 0.0444 | -0.012 | 0.1008 | 0.1226 |
|  | Hip | | 0.0689 | 0.0335 | 0.1043 | **0.0001** | 0.105 | 0.0629 | 0.1471 | **<.0001** |
|  | None(reference) | | 0 |  |  |  | 0 |  |  |  |
| Caucasian, yes | | | -0.0008 | -0.0093 | 0.0077 | 0.8457 | -0.0064 | -0.018 | 0.0052 | 0.2783 |
| BMI, kg/m2 | >=40 | | 0.0568 | 0.0384 | 0.0751 | **<.0001** | 0.0802 | 0.0317 | 0.1286 | **0.0012** |
|  | 30-40 | | 0.0209 | 0.0155 | 0.0263 | **<.0001** | 0.0207 | 0.012 | 0.0294 | **<.0001** |
|  | 25-30 | | 0.0079 | 0.0038 | 0.012 | **0.0001** | 0.0124 | 0.0059 | 0.0188 | **0.0002** |
|  | <18.5 | | -0.0103 | -0.0235 | 0.0028 | 0.1222 | 0.0505 | -0.034 | 0.1351 | 0.2417 |
|  | 18.5-25(reference) | | 0 |  |  |  | 0 |  |  |  |
| Physical Activity(kilocal/week, per 1000) | | | -0.0009 | -0.0015 | -0.0003 | **0.0024** | -0.0004 | -0.0009 | 0.0001 | 0.1496 |
| Sedentary lifestyle, hours/day | | | 0.0004 | -0.0002 | 0.0011 | 0.1997 | -0.0006 | -0.0015 | 0.0004 | 0.2385 |
| Total calcium intake (per 1200 mg/day) | | | 0.0037 | -0.0001 | 0.0076 | 0.0555 | -0.001 | -0.0068 | 0.0049 | 0.7465 |
| Total vitamin D intake (per 800 /day) | | | -0.0003 | -0.0013 | 0.0008 | 0.6372 | -0.0001 | -0.0051 | 0.005 | 0.9759 |
| MMSE score (per 3.72 point change) | | | -0.0102 | -0.0171 | -0.0032 | **0.004** | -0.0042 | -0.017 | 0.0086 | 0.5184 |
| SF-36 - Physical subscale score (per 5 units change) | | | -0.0297 | -0.0308 | -0.0286 | **<.0001** | -0.0271 | -0.029 | -0.0251 | **<.0001** |
| SF-36 - Mental subscale score (per 5 units change) | | | -0.0145 | -0.0157 | -0.0134 | **<.0001** | -0.0143 | -0.0165 | -0.0122 | **<.0001** |
| Education: University or higher | | | -0.0057 | -0.0098 | -0.0017 | **0.0053** | -0.011 | -0.0168 | -0.0051 | **0.0002** |
| Smoking | Never | | -0.0092 | -0.0151 | -0.0032 | **0.0026** | -0.0125 | -0.0226 | -0.0024 | **0.015** |
|  | Past | | -0.0048 | -0.011 | 0.0014 | 0.1265 | -0.0059 | -0.0156 | 0.0039 | 0.2379 |
|  | Current(reference) | | 0 |  |  |  | 0 |  |  |  |
| Alcohol, drinks/ day | >=3 | | -0.0111 | -0.0284 | 0.0062 | 0.2097 | -0.0004 | -0.0112 | 0.0104 | 0.9391 |
|  | 1 to 3 | | -0.007 | -0.0128 | -0.0013 | **0.0169** | -0.0004 | -0.0087 | 0.008 | 0.9336 |
|  | >0 to <1 | | -0.0075 | -0.0115 | -0.0035 | **0.0003** | 0.0043 | -0.0026 | 0.0113 | 0.2204 |
|  | None(reference) | | 0 |  |  |  | 0 |  |  |  |
| Employment | Unemployed | | 0.0032 | -0.0065 | 0.0129 | 0.5189 | 0.0205 | 0.006 | 0.035 | **0.0055** |
|  | Retired | | 0.0072 | 0.0023 | 0.0122 | **0.0043** | 0.0051 | -0.0012 | 0.0113 | 0.1109 |
|  | Homemaker | | 0.0087 | 0.0033 | 0.0141 | **0.0015** | - |  |  |  |
|  | Employed (reference) | | 0 |  |  |  | 0 |  |  |  |
| Living alone, yes | | | 0.0027 | -0.0016 | 0.007 | 0.2235 | 0.0037 | -0.0041 | 0.0116 | 0.3518 |
| BMD T-score at baseline | | | 0.0006 | -0.0015 | 0.0026 | 0.596 | 0 | -0.0039 | 0.0039 | 0.995 |
| Antiresorptive treatment at baseline , yes | | | 0.0009 | -0.0031 | 0.0048 | 0.6696 | -0.0296 | -0.0938 | 0.0345 | 0.3654 |
| Antiresorptive treatment at year 10, yes | | | 0.0016 | -0.0023 | 0.0055 | 0.4251 | 0.0218 | 0.0099 | 0.0337 | **0.0003** |
| Falls in past month, yes | | | 0.0066 | -0.0011 | 0.0142 | 0.0935 | 0.0083 | -0.0053 | 0.0219 | 0.2307 |
| Ever confined to bed (immobilized), yes | | | 0.0039 | -0.0019 | 0.0096 | 0.1896 | 0.005 | -0.0033 | 0.0134 | 0.2374 |
| Ever lost more than 10 pounds, yes | | | 0.0029 | -0.0007 | 0.0066 | 0.1177 | 0.0047 | -0.001 | 0.0104 | 0.1058 |

CFI= CaMos Frailty Index; NHNVF= non-hip non-vertebral fractures; VF = vertebral fractures;BMI= body mass index; MMSE = Mini Mental State Examination;

BMD = bone mineral density.

**Supplemental Table S23.** Worst Case Scenario 2 - imputations of the upper theoretical CFI limit for CaMos

participants who were lost-to-follow-up: Analyses in the sample **with prior fracture**.

| **Parameter** |  | |  | **Estimate** | **95% CI** | | **P-value** |
| --- | --- | --- | --- | --- | --- | --- | --- |
| Time | 10 year | | | -0.0078 | -0.0112 | -0.0044 | <.0001 |
|  | 5 year | | | 0.014 | 0.011 | 0.017 | <.0001 |
|  | Baseline (reference) | | | 0 |  |  |  |
| Age, years | >=80 | | | 0.042 | 0.0297 | 0.0543 | <.0001 |
|  | 65-80 | | | 0.0188 | 0.0147 | 0.0228 | <.0001 |
|  | 50-65 (reference) | | | 0 |  |  |  |
| Time*Age | 10 year | >=80 | | 0.032 | 0.0086 | 0.0555 | **0.0074** |
|  |  | 65-80 | | 0.0198 | 0.0144 | 0.0253 | **<.0001** |
|  |  | 50-65 | | 0 |  |  |  |
|  | 5 year | >=80 | | 0.01 | -0.0105 | 0.0304 | 0.3394 |
|  |  | 65-80 | | 0.0143 | 0.0095 | 0.019 | **<.0001** |
|  |  | 50-65 | | 0 |  |  |  |
|  | Baseline (reference) | >=80 | | 0 |  |  |  |
|  |  | 65-80 | | 0 |  |  |  |
|  |  | 50-65 | | 0 |  |  |  |
| Prevalent Fracture | NHNVF | | | 0.004 | -0.0011 | 0.0091 | 0.128 |
|  | clinical VF | | | 0.0111 | -0.0113 | 0.0335 | 0.3326 |
|  | Hip | | | 0.0258 | 0.0027 | 0.0489 | **0.0286** |
|  | None (reference) | | | 0 |  |  |  |
| Incident Fracture | NHNVF | | | 0.014 | 0.0043 | 0.0238 | **0.0048** |
|  | clinical VF | | | 0.046 | 0.0243 | 0.0677 | **<.0001** |
|  | Hip | | | 0.0744 | 0.0443 | 0.1046 | **<.0001** |
|  | None (reference) | | | 0 |  |  |  |
| Females, yes | | | | 0.0065 | 0.0025 | 0.0105 | **0.0016** |
| Caucasian, yes | | | | -0.0025 | -0.0094 | 0.0044 | 0.476 |
| BMI, kg/m2 | >=40 | | | 0.0589 | 0.0408 | 0.077 | **<.0001** |
|  | 30-40 | | | 0.0209 | 0.0163 | 0.0255 | **<.0001** |
|  | 25-30 | | | 0.009 | 0.0055 | 0.0125 | **<.0001** |
|  | <18.5 | | | -0.0075 | -0.0217 | 0.0067 | 0.3022 |
|  | 18.5-25 (reference) | | | 0 |  |  |  |
| Physical Activity(kilocal/week, per 1000) | | | | -0.0006 | -0.001 | -0.0002 | **0.0016** |
| Sedentary lifestyle, hours/day | | | | 0.0002 | -0.0004 | 0.0008 | 0.4827 |
| Total calcium intake (per 1200 mg/day) | | | | 0.0025 | -0.0007 | 0.0057 | 0.1308 |
| Total vitamin D intake (per 800 /day) | | | | -0.0003 | -0.0013 | 0.0007 | 0.5466 |
| MMSE score (per 3.72 point change) | | | | -0.009 | -0.0152 | -0.0028 | **0.0047** |
| SF-36 - Physical subscale sclore (per 5 units change) | | | | -0.0291 | -0.0301 | -0.0281 | **<.0001** |
| SF-36 - Mental subscale sclore (per 5 units change) | | | | -0.0146 | -0.0156 | -0.0135 | **<.0001** |
| Education: University or higher, yes | | | | -0.0075 | -0.0108 | -0.0042 | **<.0001** |
| Smoking | Never | | | -0.0098 | -0.015 | -0.0046 | **0.0002** |
|  | Past | | | -0.0049 | -0.0102 | 0.0003 | 0.0644 |
|  | Current (reference) | | | 0 |  |  |  |
| Alcohol, drinks/day | >=3 | | | -0.009 | -0.0177 | -0.0004 | **0.0399** |
|  | 1 to 3 | | | -0.0063 | -0.011 | -0.0015 | **0.01** |
|  | >0 to <1 | | | -0.0054 | -0.0089 | -0.0019 | **0.0023** |
|  | None (reference) | | | 0 |  |  |  |
| Employment | Unemployed | | | 0.008 | 0 | 0.016 | **0.0487** |
|  | Retired | | | 0.0071 | 0.0031 | 0.011 | **0.0004** |
|  | Homemaker | | | 0.0087 | 0.0037 | 0.0137 | **0.0006** |
|  | Employed (reference) | | | 0 |  |  |  |
| Living alone, yes | | | | 0.003 | -0.0007 | 0.0068 | 0.1137 |
| BMD T-score at baseline | | | | 0.0006 | -0.0012 | 0.0025 | 0.4966 |
| Antiresorptive treatment at baseline, yes | | | | 0.0009 | -0.003 | 0.0048 | 0.6489 |
| Antiresorptive treatment at year 10, yes | | | | 0.0038 | 0.0001 | 0.0076 | **0.0423** |
| Falls in past month, yes | | | | 0.0064 | -0.0003 | 0.013 | 0.0593 |
| Ever confined to bed (immobilized), yes | | | | 0.0035 | -0.0013 | 0.0083 | 0.1523 |
| Ever lost more than 10 pounds, yes | | | | 0.0034 | 0.0003 | 0.0065 | **0.0332** |

CFI= CaMos Frailty Index; NHNVF= non-hip non-vertebral fractures; VF = vertebral fractures;BMI= body mass index;

MMSE = Mini Mental State Examination; BMD = bone mineral density.
