# Supplementary material for: Osteoporotic fractures and obesity affect frailty progression: a longitudinal analysis of the Canadian multicentre osteoporosis study
Source: BMC Geriatr. 2018 Jan 5;18:4. doi: 10.1186/s12877-017-0692-0 (PMC5756402; doi:10.1186/s12877-017-0692-0)
Supplement: Supplementary file 2 [file 12877_2017_692_MOESM1_ESM.doc]

Table 1 The CaMos Frailty Index

	Deficit	Deficit values/description
1	Osteoarthritis	No=0, Yes=1
2	Rheumatoid arthritis	No=0, Yes=1
3	Thyroid disease	No=0, Yes=1
4	Breast cancer	No=0, Yes=1
5	Uterine cancer/prostate cancer	No=0, Yes=1
6	Inflammatory bowel disease	No=0, Yes=1
7	Hypertension	No=0, Yes=1
8	Heart attack	No=0, Yes=1
9	Stroke	No=0, Yes=1
10	Neuromuscular disease[a]	No=0, Yes=1
11	Diabetes	No=0, Yes=1
12	Kidney disease	No=0, Yes=1
13	Phlebitis/thrombophlebitis	No=0, Yes=1
14	Vision	Six levels (0, 0.2, 0.4, 0.6, 0.8, 1); descriptive range: able to see well enough to read ordinary newsprint and recognize a friend on the other side of the street, without glasses or unable to see at all=1
15	Hearing	Six levels (0, 0.2, 0.4, 0.6, 0.8, 1); descriptive range: able to hear what is said in a group conversation with at least three other people, without a hearing aid=0 to unable to hear at all=1
16	Walking	Six levels (0, 0.2, 0.4, 0.6, 0.8, 1); descriptive range: able to walk around the neighborhood without difficulty, and without walking equipment=0 to cannot walk at all=1
17	Dexterity	Six levels (0, 0.2, 0.4, 0.6, 0.8, 1); descriptive range: full use of two hands and ten fingers=0 to limitations in use of hands or fingers to requires the help of another person for all tasks, not independent even with use of special tools=1
18	Cognition	Six levels (0,0.2, 0.4, 0.6,0.8, 1); descriptive range: able to remember most things, think clearly and solve day to day problems=0 to unable to remember anything at all and unable to think or solve day to day problems=1
19	Pain	Five levels (0, 0.25, 0.5, 0.75, 1); descriptive range: free of pain and discomfort=0 to severe pain that prevents most activities=1
20	General health	Excellent=0; Very Good=0.25; Good=0.5; Fair=0.75; Poor=1
21	Change in general health (past 1 year)	Same/somewhat better/better=0; Somewhat worse/worse=1
22	Reduced daily work/other activities (last 4 weeks)	No=0, Yes=1
23	Interference with social activities due to physical/ emotional health (last 4 weeks)	Not at all/slightly=0; Moderately=0.5; Quite a
24	Limitation in moderate activities (e.g., moving table, vacuuming, golf, bowling)	bit/extremely=1 Not limited=0; Limited a little=0.5; Limited a lot=1
25	Limitation in lifting/carrying groceries	Not limited=0; Limited a little=0.5; Limited a lot=1
26	Limitation in climbing a flight of stairs	Not limited=0; Limited a little=0.5; Limited a lot=1
27	Limitation in bending, kneeling, and stooping	Not limited=0; Limited a little=0.5; Limited a lot=1
28	Limitation in bathing/dressing	Not limited=0; Limited a little=0.5; Limited a lot=1
29	Energy	Have a lot energy all/most/a good bit=0; Some of the time=0.5; a lot energy a little/none of the time=1
30	Feel tired	Feel tired a little/none of the time=0; Some of the time=0.5; Feel tired all/most/a good bit=1

[a] Parkinson's/multiple sclerosis/other

Source: Kennedy CC, Ioannidis G, Rockwood K, Thabane L, Adachi JD, Kirkland S, et al. A Frailty Index predicts 10-year fracture risk in adults age 25 years and older: results from the Canadian Multicentre Osteoporosis Study (CaMos). Osteoporos Int. 2014; 25:2825-32. Obtained with copyright permission (November 14,2017).
